# Sensitive detection of tumor mutations from blood and its application to immunotherapy prognosis

**DOI:** 10.1038/s41467-021-24457-2

**Published:** 2021-07-07

**Authors:** Shuo Li, Zorawar S. Noor, Weihua Zeng, Mary L. Stackpole, Xiaohui Ni, Yonggang Zhou, Zuyang Yuan, Wing Hung Wong, Vatche G. Agopian, Steven M. Dubinett, Frank Alber, Wenyuan Li, Edward B. Garon, Xianghong Jasmine Zhou

**Affiliations:** 1 Department of Pathology and Laboratory Medicine, David Geffen School of Medicine, University of California at Los Angeles, Los Angeles, CA USA; 2 Bioinformatics Interdepartmental Graduate Program, University of California at Los Angeles, Los Angeles, CA USA; 3 Institute for Quantitative & Computational Biosciences, University of California at Los Angeles, Los Angeles, CA USA; 4 Department of Medicine, David Geffen School of Medicine at UCLA, Los Angeles, CA USA; 5 EarlyDiagnostics Inc, Los Angeles, CA USA; 6 Department of Statistics, Stanford University, Stanford, CA USA; 7 Department of Biomedical Data Science, Stanford University, Stanford, CA USA; 8 Department of Surgery, David Geffen School of Medicine at UCLA, Los Angeles, CA USA; 9 Department of Molecular and Medical Pharmacology, David Geffen School of Medicine at UCLA, Los Angeles, CA USA; 10 VA Greater Los Angeles Health Care System, Los Angeles, CA USA; 11 Department of Microbiology, Immunology and Molecular Genetics, University of California at Los Angeles, Los Angeles, CA USA

**Keywords:** Cancer genomics, Computational biology and bioinformatics, Software, Statistical methods

## Abstract

Cell-free DNA (cfDNA) is attractive for many applications, including detecting cancer, identifying the tissue of origin, and monitoring. A fundamental task underlying these applications is SNV calling from cfDNA, which is hindered by the very low tumor content. Thus sensitive and accurate detection of low-frequency mutations (<5%) remains challenging for existing SNV callers. Here we present cfSNV, a method incorporating multi-layer error suppression and hierarchical mutation calling, to address this challenge. Furthermore, by leveraging cfDNA’s comprehensive coverage of tumor clonal landscape, cfSNV can profile mutations in subclones. In both simulated and real patient data, cfSNV outperforms existing tools in sensitivity while maintaining high precision. cfSNV enhances the clinical utilities of cfDNA by improving mutation detection performance in medium-depth sequencing data, therefore making Whole-Exome Sequencing a viable option. As an example, we demonstrate that the tumor mutation profile from cfDNA WES data can provide an effective biomarker to predict immunotherapy outcomes.

## Abstract (web summary)

It is possible to call single-nucleotide variant (SNV) in cell-free DNA (cfDNA), but the accuracy of detection is often affected by low tumour cfDNA content. Here, the authors develop a method, cfSNV, and show that it can be used even for medium-coverage whole exome sequencing of cfDNA.

## Introduction

Cell-free DNA (cfDNA) in blood has received enormous attention thanks to its clinical utility as a surrogate for tumor biopsy, especially in cases where the latter is unavailable or insufficient^1^. A tissue biopsy is invasive by nature and is only extracted from a single site. In contrast, cfDNA in blood can be obtained noninvasively, and provides a comprehensive landscape of the heterogeneous genetic alterations in tumors. Hence, a wide range of cfDNA-based applications have been developed to detect cancer^2–5^, identify the tissue of origin^4–6^, select the best therapy^7,8^, and monitor treatment^6,9–11^. All these applications depend upon an indispensable, yet underdeveloped task: precise and sensitive calling of somatic single nucleotide variants (SNV) from cfDNA sequencing data. This task is challenging to conventional SNV callers because somatic mutations in cfDNA generally have low variant allele frequency (VAF). This property follows from the major hallmarks of cfDNA: (1) cfDNA is a mixture of DNA fragments from both normal and tumor cells, and in most cancer patients the fraction of tumor-derived cfDNA is extremely low (<1% for most early-stage cancer patients^1^ and <10% even for some metastatic patients^12^). Therefore, almost all somatic mutations in tumor-derived cfDNA have much lower VAFs than in solid tumors. (2) cfDNA comes from the entire volume of a tumor and from every tumor present in a patient, so it provides complete information on clonal and subclonal mutations, while subclonal mutations generally have lower VAFs than clonal mutations.

To conquer these challenges in cfDNA data, some efforts have been made to improve experimental technologies and the computational error filtration to optimize the variant calling on targeted deep-sequencing data^13–15^. Despite the encouraging progress, existing methods are not sufficiently equipped to handle this complicated scenario, especially in medium-coverage sequencing data such as whole-exome sequencing (WES). Specifically, they are lacking in three aspects: (1) They do not automatically account for the low fraction of tumor-derived cfDNA or variability due to the tumor clonal hierarchy in the context of mutation calling, though clonality has been considered in other studies^1^. A few SNV callers (e.g., MuTect^16^) try to handle the issue of tumor impurity, but even these cannot robustly and sensitively detect mutations with VAF < 5%^16^. One mutation caller^17^ integrated clonal information to improve somatic mutation calling, but this method required extra user input of the clonal hierarchy. (2) They rely on post-filtration steps that require reliable estimation of site-level statistics (e.g., strand bias and averaged base quality). However, robust estimates are challenging to obtain for low-frequency cfDNA mutations, due to insufficient variant supporting reads. (3) They do not exploit two key features of cfDNA, namely short fragment size (~166 bp on average) and non-random fragmentation^18,19^.

In this work, we present a cfDNA SNV caller named cfSNV. It doesn’t require inputs from solid tumor samples, and it comprehensively addresses the cfDNA-specific challenges and opportunities mentioned above. Taking advantage of modern statistical models and machine learning approaches, cfSNV provides hierarchical mutation profiling and multi-layer error suppression, including error suppression in read mates, site-level error filtration and read-level error filtration. It achieves high precision and sensitivity in cfDNA samples that have both low tumor purity (tumor fraction <10%) and highly heterogeneous clonal landscapes, even for medium-coverage sequencing data such as WES, in a purely computational fashion without attachment to specially designed experimental technologies (e.g., molecular barcoding). In both simulated and real patient data, cfSNV outperforms existing tools in terms of sensitivity while maintaining high precision. With the detection performance of cfSNV, cfDNA WES data become applicable for SNV calling and related applications. As an example application, we demonstrate that applying cfSNV to cfDNA WES data yields an effective biomarker (truncal-bTMB) for immunotherapy prognosis, by simultaneously capturing both the tumor mutation burden and clonal structure information.

## Results

### cfSNV: a computational framework for calling SNVs from cfDNA

**Fig. 1:**
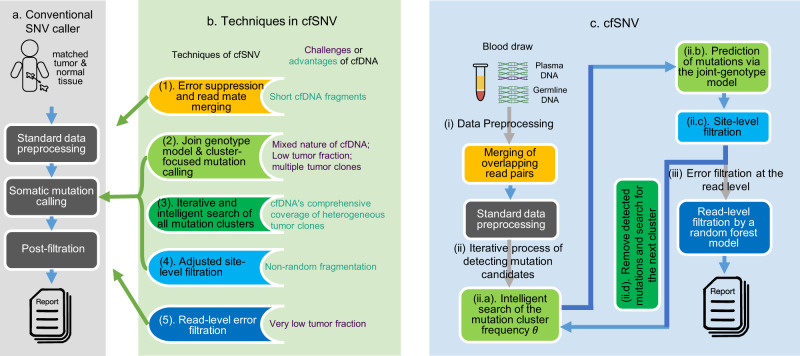
cfSNV framework and its techniques. **a** The workflow of conventional SNV callers takes the genomic data of a tumor and its matched normal tissue as inputs. **b** Five techniques introduced in cfSNV that modify the standard workflow. **c** Full workflow of cfSNV. cfSNV takes plasma DNA and germline DNA sequencing data as inputs. No tumor samples are needed. It first merges overlapping read pairs in cfDNA sequencing data. Next, we apply standard data preprocessing tools. An iterative procedure then detects mutation clusters and estimates their frequencies *θ* based on multiple, automatically selected potential mutation loci. Each iteration determines joint genotypes across sequencing regions to predict somatic SNV candidates, and masks the mutation candidates before proceeding. After all clusters and mutation candidates have been detected, a random forest classifier identifies raw read pairs with sequencing errors. Finally, somatic SNVs are reported and detected only if enough variant supporting read pairs pass the random forest screening. The background color of steps in **c** corresponds to the feature listed in **b**.

We developed the cfSNV framework (Fig. 1c) by introducing five techniques (Fig. 1b) into the standard SNV calling workflow (Fig. 1a). Each of the five techniques either overcomes a specific challenge of cfDNA or takes advantage of a specific feature of cfDNA. The challenges and features are (1) short fragments, (2) mixed nature, (3) heterogeneous clonal composition, (4) non-random fragmentation, and (5) interference from sequencing errors in low-frequency mutations (VAF < 5%). We discuss each of the challenges and features below.

cfDNA fragments usually have a short size, whose distribution peaks at 166 bp. Therefore, paired-end sequencing (usually 100 bp or 150 bp for a read) usually results in a large fraction of overlapping read mates, which can be used to suppress sequencing errors (Fig. 1b(1) and Fig. 1c(i)). This error-correction step is performed before the standard data preprocessing.

The cfDNA found in blood from cancer patients is a natural mixture that consists of a small amount of tumor-derived cfDNA among an overwhelming majority of cfDNA from normal cells. By incorporating the germline data of white blood cells (WBCs) from the same subject, we can fit a joint-genotype model that precisely describes this mixture. Specifically, we model the triplet $\left({g}_{T},{g}_{N},{g}_{W}\right)$ of genotypes, among which ${g}_{T}$ and ${g}_{N}$ actually describes the mixed nature of cfDNA by representing the genotypes of Tumor-derived cfDNA, Normal cfDNA respectively, while ${g}_{W}$ represents the genotype of the matched WBC DNA for reference purposes. The modeling of cfDNA is performed by first aggregating reads from potential mutation loci, which were identified by directly comparing the plasma cfDNA and the WBC genomic DNA data of the subject (see “Methods”). The aggregated reads across multiple potential mutation loci allows for a more robust estimate of the tumor-derived cfDNA fraction, which then serves as a parameter in the joint-genotype model for the probabilistic deconvolution of tumor-derived and normal reads at a specific locus. Note that the fraction of tumor-derived cfDNA is usually low, therefore it cannot be precisely estimated at a single locus due to the limited number of tumor-derived reads falling onto the locus.

The cfDNA from cancer patients can reflect heterogeneous clonal compositions of the tumor. Unlike tissue biopsies, a blood sample includes DNA fragments from all tumor sites, so it covers the full range of clonal and subclonal mutations^1,20^. However, heterogeneous cfDNA clonal compositions pose a great challenge to existing methods. If a statistical model fits the data from clonal mutations, it would inevitably sacrifice accuracy for subclonal mutations using the same parameters, however, this technique is in practice in all existing methods. To address this challenge, we can take advantage of the fact that the mutations associated with a given clone have similar VAF in cfDNA. The mutations are, therefore, naturally clustered according to the clonal hierarchy^1,20^. This fact permits us to develop a divide-and-conquer algorithm (Fig. 1c(ii)) that first automatically groups the mutations of the highest and similar frequencies into a cluster, then estimates parameters that best fit the data of the cluster. We then remove these detected mutations, and repeatedly perform the same operation to identify the next most frequent mutation cluster. In other words, this algorithm intelligently and iteratively searches for the best parameters of the cfDNA joint-genotype statistical model (Fig. 1c(ii.a)) to detect and model the cluster of mutations with the highest frequency in the cfDNA sample (Fig. 1c(ii.b)), then removes its loci and data. The process repeats, detecting the next most frequent mutation cluster at each iteration (Fig. 1c(ii.d)), until no more mutations are detected with confidence. Therefore, we can profile the cfDNA mutation hierarchy in terms of mutation frequencies.

Unlike the sonicated genomic DNA, the cfDNA is non-randomly fragmented. cfDNA fragments have preferred start and end positions^18^, so true mutations could cluster at certain positions on the supporting reads. Conventional tools which assume randomly fragmented genomic DNA tend to classify mutation candidates with clustered positions on reads as misalignment artifacts, therefore eliminating them^16^. Consequently, the true mutations in cfDNA samples could be removed by this artifact filter using conventional tools. We remove this artifact filter to keep true cfDNA mutations, while building a filter to jointly analyze the positions of multiple nearby mutation candidates and remove cfDNA misalignment artifacts (Fig. 1b(4) and Fig. 1c(ii.c)).

When the tumor-derived cfDNA fraction is low, sequencing errors interfere the detection of low-frequency mutations (VAF < 5%) and thus impair the detection sensitivity. We get around this problem of low signal-to-noise ratio for individual alleles by developing a machine learning approach to accurately distinguish true variants from sequencing errors for individual reads. The algorithm exploits a variety of contextual information from the region surrounding the target allele (Fig. 1b(5) and Fig. 1c(iii)) to provide an accurate prediction.

The detailed workflow is illustrated in Supplementary Fig. 1 and described in “Methods”.

### Validation of cfSNV on simulation data

**Fig. 2:**
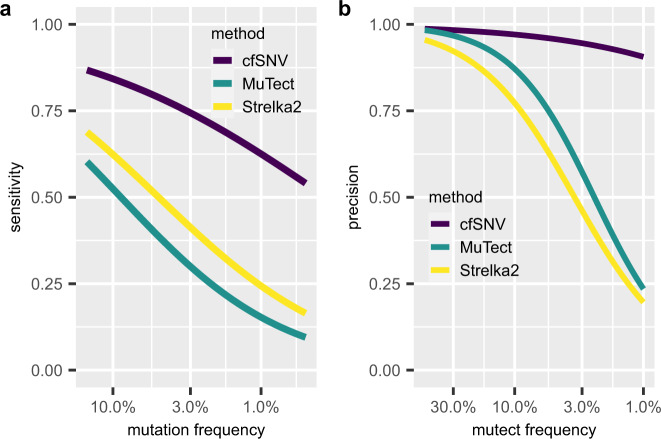
cfSNV outperforms competing methods in sensitivity and precision, especially for low-frequency mutations (VAF < 5%). **a** The sensitivity of three variant calling methods on simulation data (*n* = 6) as a function of VAF for cfSNV, MuTect, and Strelka2. Mutations were grouped based on their simulated VAF, and the sensitivity at each simulated VAF level was calculated separately. The specificity and the precision of all three methods remained at comparable and high level (Table 1). All curves were fitted using logit functions. **b** The precision of three variant calling methods on patient data (*n* = 36) as a function of VAF. Mutations detected from all samples were grouped based on their rounded VAF (two decimal places). The precision at each VAF level was estimated by the confirmation rate. The sensitivity of patient data cannot be quantified because of the unknown ground truth, but cfSNV detected the most true positive mutations. Note that MuTect has no result when VAF < 2% because its default configuration treats all mutation candidates with VAF < 2% as contamination errors, and all curves here were fitted using logit functions.

To evaluate the performance of cfSNV in calling low-frequency somatic mutations (VAF < 5%), we tested the method on simulated data, which were generated from the WES data of three healthy individuals. Specifically, we generated two WES datasets (denoted as X and Y) from one cfDNA sample collected from a healthy individual and one WES dataset (denoted as W) from the matched WBC sample from the same individual. These WES datasets X, Y, and W are used to simulate the data of two virtual cancer patients. Specifically, we first add predefined in silico somatic SNVs to the WES datasets X and Y to generate the datasets X_mutated_ and Y_mutated_, respectively. The first virtual cancer patient uses X_mutated_ and W to simulate its cfDNA WES data and WBC sample, respectively, and the second virtual cancer patient uses Y_mutated_ and W to simulate its cfDNA WES data and WBC sample, respectively (details see “Methods” and Supplementary Fig. 2). In order to simulate tumor heterogeneity, we used eight VAFs ranging from 0.5 to 15% for the predefined in silico somatic SNVs (see “Methods”). These predefined in silico somatic SNVs were the ground-truth somatic SNVs. We generated six virtual cancer patients by generating six WES datasets from cfDNA samples and three WES datasets from WBC samples from three healthy individuals and performing this simulation procedure. Mutations called at positions other than the ground-truth somatic SNVs were regarded as false positives; the detected ground-truth somatic SNVs were regarded as true positives; the undetected ground-truth somatic SNVs were regarded as false negatives. We compared cfSNV with two established SNV callers, MuTect, and Strelka2, which were designed for solid tumor tissue samples but have been utilized in studies on cfDNA samples^21,22^. Since BAMSurgeon treats read pairs as single reads, we disabled the features for overlapping read mates in the read-level filtration model. The results of the test show that cfSNV outperforms the two competing methods for all ground-truth mutations (Table 1). The performance was evaluated using sensitivity $\big(\frac{{\rm{true}}\; {\rm{positive}}}{{\rm{true}}\; {\rm{positive}}+{\rm{false}}\; {\rm{negative}}}\big)$, precision $\big(\frac{{\rm{true}}\; {\rm{positive}}}{{\rm{true}}\; {\rm{positive}}+{\rm{false}}\; {\rm{positive}}}\big)$, and false positive rate (false positive/Mb). Specifically, cfSNV achieves higher sensitivity (73.2%) than MuTect (34.6%) and Strelka2 (44.1%), while maintaining high precision (94.0% vs. 87.7% and 90.6%, respectively) and low false positive rates (1.10 vs. 1.15, and 1.07, respectively). When looking at low-frequency mutations (VAFs < 5%) specifically, the advantage of cfSNV over other methods is more evident (Table 2 and Fig. 2a): cfSNV detected 3.58 and 2.44 times more mutations than MuTect or Strelka2. We also generated simulation data at a higher sequencing depth (around 2200×) using a pooled cfDNA sample (see “Methods”). On this dataset, cfSNV showed an even higher increase in performance compared to the two competing methods (see Supplementary Table 1 and Supplementary Table 2).

**Table 1:** Validation performance of cfSNV on simulation data.

Performance metrics	cfSNV	MuTect	Strelka2
# predicted positives	7725	3915	4818
# true positives	7260	3432	4367
# false positives	465	483	451
Precision	93.98%	87.66%	90.60%
Sensitivity	73.24%	34.60%	44.10%
False positive rate (1/Mb)	1.10	1.15	1.07

In the simulated dataset, 9912 mutations were inserted in silico as ground truth somatic mutations.

**Table 2:** Sensitivity and precision of cfSNV for mutations with different VAFs on simulation data.

VAF for simulated mutations	# Ground-truth mutations	cfSNV		MuTect		Strelka2	
		Sensitivity (# true positive)	Precision (# false positive)	Sensitivity (# true positive)	Precision (# false positive)	Sensitivity (# true positive)	Precision (# false positive)
0.5%	1958	60.78% (1190)	99.17% (10)	16.65% (326)	89.81% (37)	23.9% (468)	97.30% (13)
1%	1970	61.52% (1212)	91.20% (117)	17.06% (336)	76.36% (104)	27.36% (539)	97.82% (12)
3%	1035	66.28% (686)	85.54% (116)	19.42% (201)	62.04% (123)	25.12% (260)	80.00% (65)
5%	978	73.31% (717)	89.85% (81)	29.75% (291)	79.73% (74)	41.62% (407)	87.90% (56)
8%	967	79.21% (766)	94.45% (45)	42.40% (410)	93.82% (27)	56.57% (547)	95.30% (27)
10%	1010	86.34% (872)	97.54% (22)	54.75% (553)	96.68% (19)	68.91% (696)	98.86% (8)
13%	1027	90.46% (929)	98.41% (15)	62.71% (644)	97.13% (19)	72.64% (746)	97.77% (17)
15%	967	91.83% (888)	93.77% (59)	69.39% (671)	89.35% (80)	72.8% (704)	73.56% (253)
Total	9912	73.24% (7260)	93.98% (465)	34.62% (3432)	87.66% (483)	44.06% (4367)	90.64% (451)

### Validation of cfSNV on patient data

Next, we tested the ability of cfSNV to call somatic mutations on patient data. We collected WES data of samples obtained from six metastatic prostate cancer (castrate-resistant prostate cancer, CRPC) and twelve metastatic breast cancer (MBC) patients^21^ (“Methods”). For each patient, we used the WES data from a metastatic tumor biopsy sample, a WBC sample, and two plasma cfDNA samples. The cfDNA samples were drawn at two different time points after the patients were diagnosed as metastatic, with time gaps in the range 14–138 days (Supplementary Table 3). We compare the different SNV callers in terms of the confirmation rate, defined as the fraction of mutations detected in one cfDNA sample that are also confirmed to be present in either the matched tumor tissue or the other cfDNA sample. Following a recent study^21^, we confirm the presence of a mutation by the number of variant supporting reads from the raw sequencing data (i.e., supported by $\ge 3$ variant reads, see “Methods”)^21^. The confirmed mutations in the matched tumor tissue are regarded as true positives. As a single tumor biopsy sample cannot profile all tumor clones in a metastatic cancer patient, we also regarded mutations present in both plasma samples but absent in the tumor biopsy as true positives. Thus, when we count confirmed mutations in either the tumor biopsy or the other cfDNA sample, this confirmation rate is basically the same as the precision on the patient data. We performed the evaluation in the following two steps.

First, we tested the confirmation rate of cfSNV across different samples. We applied cfSNV to the 18 cfDNA samples of the timepoint 1 (T1) to obtain a baseline mutation set for calculating the confirmation rate. We validated the truncal and branch mutations detected. A mutation is defined as truncal if its VAF is above 60% of the average VAF of the five most frequent mutations in the sample; otherwise, it is branch (“Methods”). Averaged across all 18 subjects, 97.8% and 77.5% of truncal mutations are confirmed in the cfDNA sample from the timepoint 2 (T2) and the tumor biopsy of the same subject, respectively. 93.3% and 62.4% of branch mutations are confirmed in the T2 cfDNA sample and the tumor biopsy of the same subject respectively (Supplementary Fig. 3). The confirmation rates are similar if we instead use mutations detected in the 18 T2 cfDNA samples as a baseline (Supplementary Fig. 3, 96.6% and 78.8% for truncal mutations, 93.2% and 60.2% for branch mutations in the T1 cfDNA sample and the tumor biopsy respectively). We observed that the larger the time gap between the two blood draws, the lower the confirmation rate of branch mutations between the two cfDNA samples (Pearson’s correlation between the time gap and the confirmation rate = –0.49 (95% confidence interval (CI) = [−0.71, −0.19]), two-sided Pearson’s correlation test *p* = 0.0024, *t* statistic = −3.28, degree of freedom (df) = 34, see Supplementary Fig. 4 and Supplementary Table 3). This trend was not observed for truncal mutations (Pearson’s correlation between the time gap and the confirmation rate = −0.06 (95% CI = [−0.38, 0.27]), two-sided Pearson’s correlation test *p* = 0.721, *t* statistic = −0.36, df = 34, see Supplementary Fig. 4 and Supplementary Table 3). This observed trend implied that the mutation landscape of cfDNA changed with time, especially for branch mutations.

**Fig. 3:**
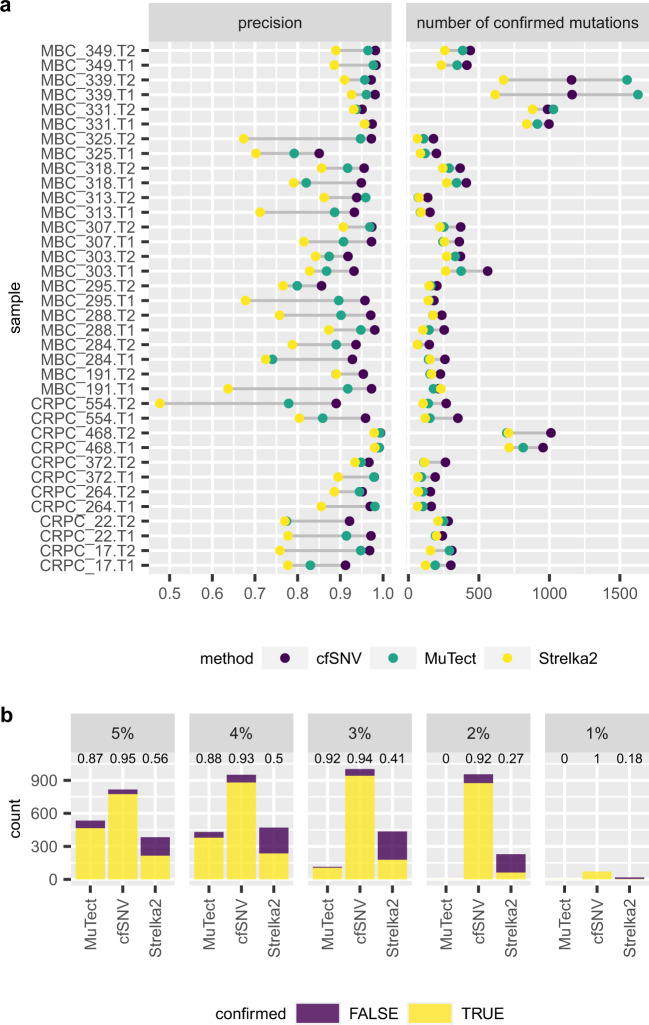
Somatic SNV calling on cfDNA sequencing samples from cancer patients. **a** Total number of confirmed mutations and precision using cfSNV, MuTect, and Strelka2. The precision is the number of confirmed mutations (in either the tumor biopsy or the plasma sample) divided by the total number of detected mutations. In the sample name, T1 and T2 indicate the first time point and the second time point of blood plasma samples respectively. **b** The total number of low-frequency variants and their confirmation status found by cfSNV, MuTect and Strelka2 from all plasma samples. Low-frequency variants are divided into five groups according to their rounded VAF, and the number of confirmed and unconfirmed mutations for each variant group are plotted in five subfigures for comparing between our method and two competing methods. The number at the top of each bar indicates the precision.

Second, we compare cfSNV with competing methods (MuTect and Strelka2) on the same samples in terms of the precision, i.e., the confirmation rate that counts the confirmed mutations in either the tumor biopsy or the other plasma sample. Although these metastatic plasma samples with high tumor fractions (ranging from 13 to 79%) are not the best scenario to demonstrate the power of cfSNV (as the majority of mutations have VAF > 10%, see Supplementary Fig. 5), still cfSNV outperformed both methods, achieving the highest precision in 33 out of 36 samples (Fig. 3a). For the remaining 3 samples, cfSNV’s precision was only marginally lower than the highest precision (by 0.2%, 1.1%, and 2.1%). Note that the high tumor fraction of these samples indicated the overall high level of tumor-derived cfDNA among all cfDNA, but branch mutations come from minor tumor clones and can have low VAF. Below we specifically evaluated the three methods on mutations with low VAF (<5%). In fact, the lower the VAF of a mutation, the more power cfSNV exhibits compared to other methods (Fig. 2b). At a VAF of 1%, cfSNV yielded 82.4% higher precision and identified 23.7 times more confirmed mutations than Strelka2 (Figs. 3b and  2b); no results are available for MuTect here because its default configuration treats all mutation candidates with VAFs <2% as contamination errors. At a VAF of 3%, cfSNV yielded 1.8% and 53.0% higher precision and identified 9.0 and 5.3 times more confirmed mutations than MuTect and Strelka2, respectively (Figs. 3b and  2b). At a VAF of 5%, cfSNV yielded 7.8% and 38.7% higher precision and identified 1.7 and 3.6 times more confirmed mutations than MuTect and Strelka2, respectively (Figs. 3b and  2b). For the low-frequency mutations (VAF < 5%), cfSNV showed stronger advantage toward MuTect and Strelka2 on the patient data than on the simulation data. The performance was contributed by the higher sequencing depth at these mutations in the patient data (mean depth 708x), and the application of the features on overlapping read mates in the read-level filtration model. Across all VAF ranges, on average cfSNV yielded 4.6% and 13.3% higher precision (Fig. 3a) and detected 1.5 and 1.8 times more confirmed mutations (Fig. 3a) than MuTect and Strelka2, respectively, demonstrating an overall higher precision and sensitivity.

Note that all three methods have consistently higher confirmation rates in the second plasma sample than the matched tumor tissue sample, implying that plasma cfDNA offers a more comprehensive coverage of tumor mutations than a single tumor biopsy for metastatic cancer patients. Therefore, whenever multifocal sampling of tumors from a metastatic cancer patient is infeasible, cfDNA is a viable alternative to obtain comprehensive mutation profiles.

### Experimental analysis of five techniques

Here, we quantitatively assess how each of the five techniques impacts the performance of cfSNV.

Taking advantage of the overlapping read mates induced by the short cfDNA fragments, we suppressed sequencing errors. The paired-end sequencing of cfDNA results in significant overlaps in the read mates. For example, in 95% of 59 cfDNA samples collected from Adalsteinsson et al.^21^, >50% of read mates overlap (2 × 100 bp paired-end sequencing, Supplementary Fig. 6a and Supplementary Table 4); in all of the 30 cfDNA samples from a cohort of NSCLC patients, >50% of read mates overlap (2 × 150 bp paired-end sequencing, Supplementary Fig. 6b). Our result shows that using overlapping read pairs, combined with a machine learning approach (Fig. 1b(5)), can facilitate the detection of true mutations while rejecting sequencing errors. Specifically, we compare the models with and without using the overlapping read information, the area under curve (AUC) of receiver operating characteristics (ROC) averaged across 36 independent test datasets (cfDNA samples from Adalsteinsson et al.^21^) shows significant improvement (one-sided Wilcoxon rank-sum test *p*-value = 5.187e − 06, W statistic = 256, Supplementary Fig. 6c, d).

**Fig. 4:**
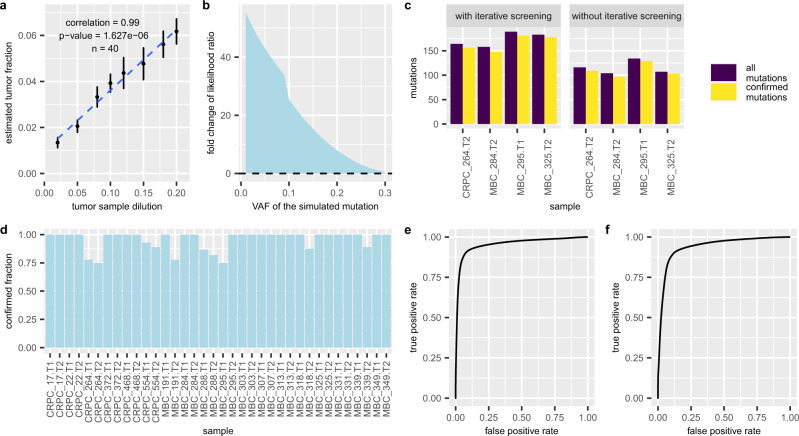
Experimental analysis of five techniques. **a** Performance of mutation cluster frequency estimation in terms of the correlation between the estimated tumor fraction and the true dilution ratio. This experiment uses simulated data based on WES of a single patient, with dilution ratios ranging from 2 to 20%. The points and the error bars are presented as mean ± s.d. of independently generated datasets (*n* = 5) at each dilution. The *p*-value was calculated from two-sided Pearson’s correlation test: Pearson’s correlation = 0.99 (95% CI = [0.95, 1.00]), two-sided Pearson’s correlation test *p*-value = 1.627e−06 (*t* statistic = 9.1154, df = 57), *n* = 40. **b** the fold change in the likelihood ratio between cfSNV models with and without a step to estimate the mutation cluster frequency, based on simulated mutations at different VAFs. **c** Number of confirmed mutations and all mutations detected with and without the iterative screening procedure. **d** Confirmation rate of rescued mutations after adjusting conventional site-level post-filtration. **e**, **f** Performance of read-level variant classifier on independent testing data. **e** The averaged receiver operating characteristic curve (ROC) of applying the classifier to labeled data taken from 24 cfDNA sequencing samples of 12 metastatic breast cancer patients. **f** The averaged ROC of applying the classifier to labeled data taken from 12 cfDNA sequencing samples of 6 metastatic prostate cancer patients.

Considering the mixed nature of cfDNA, we enhanced mutation detection by a joint-genotype model that allows for cluster-oriented mutation calling. As aforementioned, a model cannot use the same parameter to best fit both clonal and subclonal mutations that have distinct VAFs. We, therefore, introduce the divide-and-conquer strategy to first train the model to detect only mutations of the cluster with the highest frequency, then remove loci of these detected mutations, and repeat the same procedure for the next most frequent mutation cluster. The key component of this iterative process is our joint-genotype model that supports the cluster-oriented mutation calling in a single iteration. Specifically, the model has a parameter of describing how frequent the mutation cluster is (denoted as $\theta$) and this parameter allows the model to best fit the data of only those mutations in this cluster, not all the mutations of the heterogeneous landscape. Therefore, we assess the model by answering two questions: (1) Can $\theta$ estimated by our method reflect the VAFs of the mutations in the most frequent mutation cluster? We designed three experiments to answer this question, using simulated data with synthetic mutations, simulation data obtained by mixing real sample data with a known dilution ratio, and real cfDNA data. In the first experiment, we generated five independent simulation datasets by randomly inserting three groups of synthetic mutations into the WBC data from an MBC patient: one mutation cluster with a VAF of 20%, one cluster with a VAF of 8%, and one with a VAF of 2% (“Methods”). For all five datasets, our method not only automatically identifies the most frequent cluster and estimates its VAF, but also finds the other two clusters in subsequent iterations (Supplementary Fig. 7a). In the second experiment, we subsampled and mixed sequencing reads from the WBC and the primary tumor samples, both taken from the same cancer patient (“Methods”). The two samples were mixed at eight varied concentrations (2% to 20%) and five independent mixtures are generated at every concentration. The tumor fraction, which is estimated by the frequency $\theta$ of the most frequent mutation cluster in these mixed samples, correlates strongly (Pearson’s correlation = 0.99 (95% CI = [0.95, 1.00]), two-sided Pearson’s correlation test *p*-value = 1.627e−06 (*t* statistic = 18.464, df = 6), *n* = 40) with the ground-truth mixing dilution (Fig. 4a) across the study population. In the third experiment, we used data from two independent sequencing experiments (WES and whole-genome sequencing (WGS)) on the same cfDNA sample in each of the 41 cancer patients (in total 59 plasma samples). Specifically, we compared the tumor fraction estimated by cfSNV in WES to that estimated by ichorCNA^21^ in WGS. This result, shown in Supplementary Fig. 7b, also confirms that our method accurately estimates the frequency of the major mutation clusters. (2) Does accurately estimating the mutation cluster frequency $\theta$ enhance mutation detection? We generated simulated sequencing data with a list of predefined $\theta$ values, from 0 to 100%, and found the optimal $\theta$ that fits the joint-genotype model. Our performance metric is the model-to-data fitness ratio, defined as the ratio between the likelihoods of correct and incorrect joint genotypes (“Methods”). A higher ratio means that the model is a better fit, so the mutation is more likely to be identified. Our result shows that any given mutation is best fit by the model when $\theta$ takes on a value close to the mutation’s frequency (Supplementary Fig. 7c–f). In addition, when comparing the fitness of the model with and without $\theta$ (i.e., comparing the two likelihood ratios), we find that the smaller a mutation’s VAF, the larger the difference (e.g., the model-to-data fitness ratio is 40 times higher with $\theta$ present, for VAF < 5%). This relationship indicates that an accurate $\theta$ estimate enhances the detection power for low-frequency mutations (VAF < 5%, Fig. 4b). Furthermore, we used four cfDNA samples whose tumor fraction is below 20% to further confirm this conclusion (Supplementary Fig. 7g). More mutations were detected when the assigned $\theta$ approached the true value of the mutation cluster frequency.

Considering the heterogeneous clonal landscape in tumor-derived cfDNA, we enhanced the sensitivity of mutation detection by an iterative process. With mutation detection enhanced by the joint-genotype model in every iteration, we assessed the impact of the iterative procedure on the mutation detection. We compared two versions of cfSNV, with and without the iterative process, on real data: four cfDNA samples whose tumor fraction is below 20%. With the iterative process, cfSNV detected 1.40–1.73 times more confirmed mutations (true positives) than cfSNV without the iterative process (Fig. 4c). Both versions had high precision: namely, 95.4% and 94.9% for cfSNV with and without the iterative process, respectively (Fig. 4c).

We designed post-filtration to accommodate the non-random fragmentation of cfDNA and achieved high confirmation rate in the rescued mutations. Compared with the conventional post-filtration strategy, which models the distribution of variant-base positions on reads, our filtration strategy rescues 1–15 mutations (6.9 on average) per sample among the 36 plasma samples from the 12 MBC and 6 CRPC patients in this study. In 69.4% (26) of the samples, 100% of the rescued mutations are confirmed in either the matched tumor biopsy or the other plasma sample (Fig. 4d).

As the tumor fraction content is low in cfDNA, we utilized a machine learning approach to to distinguish true mutations from sequencing errors in cfDNA reads. The independent data used to test the machine learning model are data from the 12 MBC and 6 CRPC patients. We hand-labeled read pairs containing high-confidence mutations or sequencing errors and applied the random forest classifier (“Methods”). Our method achieves an average area under receiver operating characteristic curve (AUC-ROC) of 0.95 over the MBC cfDNA samples (Fig. 4e and Supplementary Fig. 8) and an average AUC-ROC of 0.94 over the CRPC cfDNA samples (Fig. 4f and Supplementary Fig. 8). This result shows that our machine learning model can distinguish true mutations from sequencing errors with high accuracy at the level of individual reads. It implies that our machine learning model is non-specific to tumor types and can be generalized to include samples from many kinds of tumors.

### Application to predict the outcome of anti-PD-1 treatment

Cancer immunotherapies, which activate a patient’s own immune system to kill tumor cells, have remarkably improved the clinical outcome of a subset of patients with non-small-cell lung cancer (NSCLC)^23^. To better predict the therapy response and identify patients with potential clinical benefit, tumor mutational burden (TMB) based on solid tumor biopsies, which measures the extent of nonsynonymous genetic changes of the tumors, has been studied and utilized as a biomarker in various cancer types^23–25^, including NSCLC. In addition to the work on TMB, recent studies^8,26^ have shown that blood-based tumor mutational burden (bTMB) is an attractive alternative to tissue-based TMB due to three advantages: (1) noninvasiveness, (2) more comprehensive mutation coverage (by cfDNA) than a single-site tumor biopsy, and (3) the VAFs of mutations in cfDNA reflect their clonality in tumors. It has also been shown that in solid tumor samples, high truncal neoantigen load and low intra-tumor heterogeneity more significantly associate with longer progression-free survival (PFS) than total neoantigen load alone^27,28^. Advantage (3) allows the inference of the clonality of tumor-derived mutations from cfDNA, and thus improves the prognosis. To fully exploit advantages (2) and (3), profiling of cfDNA with a broad genomic coverage (e.g., whole exome) is needed. However, due to the lack of efficient tools to accurately call SNV from cfDNA using medium-coverage WES data (e.g. 200×), all current bTMB methods^8,26^ use small gene panels (<600 genes) in order to perform deep sequencing (e.g., >5000×). Small panels can only sparsely sample the total mutation landscape, so the resulting estimates of TMB or bTMB are influenced by population and sampling variation^29^. In contrast, cfSNV enables sensitive and precise mutation profiling in even medium-depth sequencing data, thus allowing us to fully profile the mutation landscape as well as benefit from all the other advantages offered by cfDNA. Specifically, we exploit the clonality information in cfDNA to develop a immunotherapy prognosis metric, truncal-bTMB, which uses only truncal mutations called by cfSNV from the WES profiling of cfDNA samples (“Methods”). We applied this metric to predict the outcomes of anti-PD-1 treatment, and achieved better performance compared to bTMB and TMB.

**Fig. 5:**
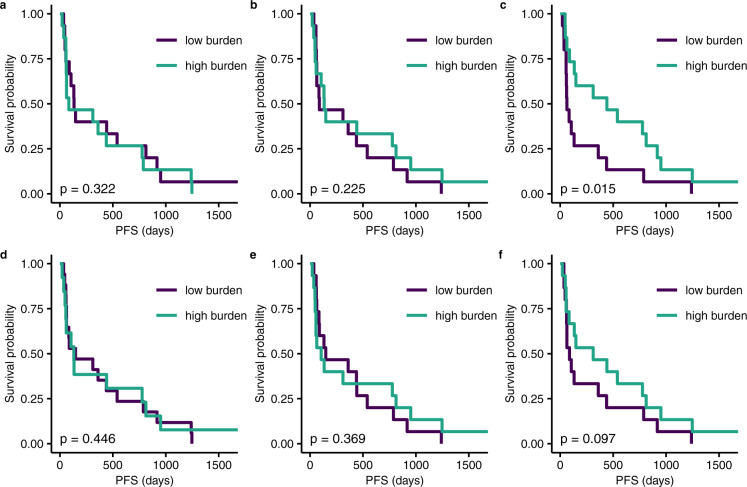
Kaplan–Meier curves for progression-free survival (PFS) on the pre-treatment cfDNA sequencing data of 30 advanced NSCLC patients. **a**–**c** Kaplan–Meier curves based on truncal-bTMB calculated using MuTect, Strelka2, and cfSNV. The high-burden and low-burden groups in each plot are defined by the median value of the measure: MuTect (**a**, hazard ratio (HR) = 0.839, 95% CI [0.403, 1.747], Z statistic = −0.48), Strelka2 (**b**, HR = 0.745, 95% CI [0.352, 1.581], Z statistic = −0.76), or cfSNV (**c**, HR=0.438, 95% CI [0.205, 0.938], Z statistic = −2.07). **d**–**f**, Kaplan–Meier curves based on bTMB calculated using MuTect, Strelka2, and cfSNV. The high-burden and low-burden groups in each plot are defined by the median value of the measure: MuTect (**d**, HR = 0.948, 95% CI [0.451, 1.990], Z statistic = −0.14), Strelka2 (**e**, HR = 0.883, 95% CI [0.415, 1.880], Z statistic = −0.33), or cfSNV (**f**, HR = 0.611, 95% CI [0.288, 1.295], Z statistic = −1.29). All *p*-values were calculated from one-sided log-rank test. There is no multiple testing adjustment.

To comprehensively evaluate the predictive power of the measures bTMB and truncal-bTMB (facilitated by cfSNV), we studied a cohort of 30 NSCLC patients who received anti-PD-1 treatment (pembrolizumab). Blood samples were drawn from these patients before their treatment. All cfDNA samples were sequenced with WES. First, we compared bTMB based on different mutation callers (MuTect, Strelka2, and cfSNV). We split the 30 patients into two groups using the population median^23^ of the respective truncal-bTMB metric (the distribution shown in Supplementary Fig. 9), which we call the high-burden ($>$median) and low-burden ($\le$median) groups, and evaluate how Kaplan–Meier survival curves of the progression-free survival time (PFS) differ between the two groups. The truncal-bTMB calculated based on cfSNV mutation calls had the most significant one-sided log-rank test *p*-value (Fig. 5a–c), 0.015 (cfSNV, Z statistic = −2.07) vs. 0.225 (Strelka2, Z statistic = −0.76) and 0.322 (MuTect, Z statistic = −0.48), implying that the truncal-bTMB derived from cfSNV has the highest power for predicting patients with longer PFS. We further show that the truncal-bTMB metric is a more powerful predictor than the bTMB metric, for which the PFS association is less significant (Fig. 5d–f), one-sided log-rank test *p*-value 0.097 (cfSNV, Z statistic = −1.29) vs. 0.369 (Strelka2, Z statistic = −0.33) and 0.446 (MuTect, Z statistic = −0.14), although cfSNV mutation calls again yielded the best predictor. Note that using any of the three callers, truncal-bTMB always offers better predictive power than bTMB, indicating that combining mutation clonality and intra-tumor heterogeneity improves predictive power. Interestingly, comparing the three variant calling methods, the disagreement of the high/low burden group assignment concentrated on the samples with estimated tumor fraction lower than 20%, indicating that those samples contributed most to the better performance of cfSNV. This is consistent with the aforementioned major strength of cfSNV in sensitively and precisely calling mutations in samples with low tumor fraction. Furthermore, we compared tumor-derived TMB with bTMB and truncal-bTMB on a subset of 14 patients, for whom the tumor biopsies were available. Again, cfSNV-derived truncal-bTMB had the best performance in predicting outcomes (Supplementary Fig. 10a–c) also in this cohort, where the one-sided log-rank test *p*-values are 0.028 for truncal-bTMB, 0.280 for TMB, and 0.067 for bTMB with cfSNV, respectively. In this cohort, cfSNV-derived truncal-bTMB showed the best performance in predicting the PFS outcome, as the truncal-bTMB values gave the most significant *p*-value between the high-burden group and the low burden group. From the survival analysis, the high truncal-bTMB in the plasma cfDNA was associated with the improved progression-free survival. Therefore, by exploiting the unique advantages of cfDNA using cfSNV, our proposed measure provides an effective prognosis indicator for anti-PD-1 immunotherapy on NSCLC patients.

## Discussion

We presented a computational framework, cfSNV, that sensitively detects low-frequency somatic SNVs (VAF < 5%) in cfDNA sequencing data. cfSNV is equipped with a series of techniques to address cfDNA-specific challenges (i.e., mixed tumor-derived/normal cfDNA, low tumor-derived cfDNA fraction, and high heterogeneity) and take advantage of cfDNA-specific features (high rate of overlapping reads, complete coverage of the mutation landscape, and non-random fragmentation). Specifically, (1) we designed a joint-genotype statistical model, parametrized by the mutation cluster frequency, to probabilistically deconvolute the mixture of tumor-derived and normal reads in cfDNA data; (2) we developed an iterative approach to detect clusters of mutations with different VAFs; (3) we designed a data preprocessing step that exploits the overlapping read mates caused by short cfDNA fragments to improve data quality; (4) we developed a procedure for filtering misalignment errors that accounts for the non-random fragmentation pattern of cfDNA; and (5) we developed a machine learning approach that incorporates the sequencing context to filter errors at the level of individual reads.

Equipped with these techniques and special considerations for cfDNA, we have shown cfSNV outperforms the existing methods in terms of overall precision and sensitivity. The cancer patients of this study are metastatic, so their plasma cfDNA has high fractions of tumor-derived cfDNA and carries many high-frequency mutations that can be usually detected by all conventional methods. For these high-frequency mutations, cfSNV can still achieve the best performance. Especially, for those low-frequency mutations, cfSNV achieves higher sensitivity than competing methods, without sacrificing precision, not only in the real patient data but also in the simulation data. These results demonstrate that cfSNV could provide high-quality discovery of both low- and high-frequency mutations even in medium-depth sequencing data, such as WES data.

cfSNV is a general computational framework, applicable to medium- or deep-coverage cfDNA sequencing data. While the existing methods address the challenge of low tumor-content in cfDNA by ultra-deep sequencing of a limited number of loci, the power of cfSNV reduces the required sequencing depth, therefore making cfDNA WES applicable for SNV calling and related applications. Here we presented an application that offers an effective immunotherapy response predictor (truncal-bTMB) by exploiting the comprehensive coverage of the clonal mutation landscape in cfDNA. Note that our method requires sequencing of the matched WBC sample. This requirement, though incurring additional costs, is essential for reducing the impact of clonal hematopoiesis of indeterminate potential (CHIP), and therefore has become common practice^30,31^. We believe that cfSNV will facilitate cfDNA-based therapy prognosis and longitudinal monitoring.

## Methods

### Data collection

We collected WES data of 42 metastatic cancer patients from two sources: (1) the data of 41 patients were obtained from Adalsteinsson et al.^21^ under dbGaP accession code phs001417.v1.p1. Each patient’s data includes a WBC sample, a tumor biopsy sample, and one or two plasma cfDNA samples. Among the 41 patients, 18 have two plasma cfDNA samples. One patient (MBC_315) had her cfDNA sample sequenced with both WES and deep WGS. (2) The data of one patient was obtained from Butler et al^22^. (European Nucleotide Archive accession numbers ERS700858, ERS700859, ERS700860, and ERS700861). The data include a WBC sample, a primary breast cancer biopsy sample, a metastatic liver biopsy sample, and a plasma cfDNA sample. We also collected samples from 30 NSCLC patients and 3 healthy individuals and generated our own WES data as described below.

### Human subjects

The plasma samples, tumor biopsy samples and WBC samples from 30 NSCLC patients were previously collected at University of California, Los Angeles for KEYNOTE-001^32^ and KEYNOTE-010^33^, under clinical trial registration ﻿ClinicalTrials.gov number NCT01295827 and NCT01905657. All patients provided written consent before any study-related procedures were performed. The WBC samples and the tumor biopsy samples were collected from each patient at the start of the treatment. The plasma samples were collected from each patient at 0-week, 6-week, and 12-week, measured at the start of the treatment. The samples were excluded if the duplication rate was >80% in the sequencing data. In total, 16 of 30 tumor biopsy samples were excluded. We also purchased plasma samples and WBC samples of 3 healthy individuals from Biopartners, Inc. (Woodland Hills, CA). The 3 healthy individuals have provided informed consent for research use. The project was approved by the Institutional Review Board (IRB) of University of California, Los Angeles (IRB# 12-001891, IRB# 11-003066, and IRB# 13-00394).

### Genomic DNA WES library construction

For the 30 NSCLC patients, the WBC samples and the tissue samples underwent multiplexed paired-end WES to a target depth of 100–150× on HiSeq 2000/3000 (Illumina, San Diego, CA) performed by the UCLA Technology Center for Genomics & Bioinformatics. Macrodissection was not performed. DNA isolation was performed with DNeasy Blood & Tissue Kit (Qiagen, Germantown, MD); exome capture and library preparation were performed with the KAPA HyperPrep Kit and Nimblegen SeqCap EZ Human Exome Library v3.0 (Roche, Switzerland) according to the manufacturer’s protocol. For the three healthy individuals, the WBC gDNA isolation was performed with DNeasy Blood & Tissue Kit (Qiagen) and sonicated by Covaris system (Woburn, MA). Ampure XP beads (Beckman-Coulter, Atlanta, GA) size selection was further performed to enrich the fragments between 100 and 250 bp. The gDNA WES library was constructed with the SureSelect XT HS kit from Agilent Technologies (Santa Clara, CA) according to the manufacturer’s protocol. No molecular barcodes were used in the sequencing libraries. In brief, 100 ng of gDNA was used as input material. After end repair/dA-tailing of cfDNA, the adaptor was ligated. The ligation product was purified with Ampure XP beads and the adaptor-ligated library was amplified with index primer in 7-cycle PCR. The amplified library was purified again with Ampure XP beads, and the amount of amplified DNA was measured using the Qubit 1xdsDNA HS assay kit (ThermoFisher, Waltham, MA). 1000 ng of DNA sample was hybridized to the capture library and pulled down by streptavidin-coated beads (ThermoFisher). After washing the beads, the DNA library captured on the beads was re-amplified with 10-cycles of PCR. The final libraries were purified by Ampure XP beads. The library concentration was measured by Qubit. The library quality check was further performed with Agilent Bioanalyzer before the final step of 2 × 150 bp paired-end sequencing by Genewiz (South Plainfield, NJ).

### Plasma cfDNA WES library construction

For each of the 30 NSCLC patients, venipuncture was performed by trained phlebotomists such as nurses or medical assistants. Blood tubes were centrifuged at 1800 × *g* for 20 min at room temperature and plasma supernatant was isolated within 2 h of collection. The 30 NSCLC patients and the three healthy individuals’ plasma samples were stored at −80 °C until use. Then, cfDNA was extracted from their plasma samples using the QIAamp circulating nucleic acid kit from QIAGEN (Germantown, MD). The cfDNA WES library was constructed with the SureSelect XT HS kit from Agilent Technologies (Santa Clara, CA) according to the manufacturer’s protocol. No molecular barcodes were used in the sequencing libraries. In brief, 10–15 ng of cfDNA was used as input material. After end repair/dA-tailing of cfDNA, the adaptor was ligated. The ligation product was purified with Ampure XP beads (Beckman-Coulter, Atlanta, GA) and the adaptor-ligated library was amplified with index primer in 10-cycle PCR. The amplified library was purified again with Ampure XP beads, and the amount of amplified DNA was measured using the Qubit 1xdsDNA HS assay kit (ThermoFisher, Waltham, MA). 700–1000 ng of DNA sample was hybridized to the capture library and pulled down by streptavidin-coated beads. After washing the beads, the DNA library captured on the beads was re-amplified with 10-cycles of PCR. The final libraries were purified by Ampure XP beads. The library concentration was measured by Qubit, and the quality was further examined with Agilent Bioanalyzer before the final step of 2x150bp paired-end sequencing by Genewiz (South Plainfield, NJ), at an average coverage of 200x.

### The workflow of cfSNV

cfSNV takes the plasma cfDNA and germline DNA sequencing data of a patient as inputs and detects SNVs using the three-step process described below (Supplementary Fig. 1). The outputs at the end of the pipeline are the detected mutations and the tumor fraction.

(i) Data preprocessing. A short cfDNA fragment (peak size 166 base pairs) usually has overlapping read mates when using paired-end sequencing data which can lead to double-counting overlapping regions and bias VAFs. Simply discarding overlapping regions^16,34,35^ would waste a large amount of sequencing data. Actually, these overlapping regions provide the opportunity to detect and suppress sequencing errors as two copies of the original DNA template are available. Therefore, in addition to the standard data preprocessing steps of alignment, deduplication, local realignment, and base quality recalibration, we perform an additional step: merging overlapping read mates. This step is performed before the standard preprocessing pipeline (Supplementary Fig. 1) to address the two challenges and utilize the overlapping regions. This step corrects the read counts in overlapping regions, thereby removing the bias in VAFs caused by double-counting, and also detects sequencing errors by comparing the context of the forward read and the reverse read in the overlapping region. Specifically, inconsistent bases in the overlapping region are corrected to be the base call with higher quality, while consistent bases are confirmed and assigned a high base quality. This step is implemented by FLASh^36^. Those read mates that are overlapping are merged as single-end reads, while the rest of read pairs are treated as paired-end reads. The parameters for FLASh were adjusted to accommodate the typical fragment lengths of cfDNA and read lengths in sequencing data. We aligned paired-end reads and single-end reads separately to the hg19 human reference genome. We used bwa mem^37^ to align the reads, and samtools^38^ to sort them. Then we used picard tools^39^ MarkDuplicates to remove duplicate reads resulting from PCR amplification. After this step, we added read group information to the bam file using picard tools AddOrReplaceReadGroups, and realigned reads around indels using GATK^34,35^ with Java^40^. The target regions in realignment were identified through GATK RealignerTargetCreator, then reads around target regions were realigned using GATK IndelRealigner. Finally, base quality scores were recalibrated using GATK BaseRecalibrator and PrintReads.

(ii) Iterative process of detecting mutation candidates. As illustrated in Supplementary Fig. 1, this process repeats a sequence of four steps until no more mutation candidates are detected with confidence. In each complete iterative round, a mutation cluster is determined.

(Step 1) Estimating the mutation cluster frequency $\theta$ of the most frequent mutation cluster. As the frequency of mutations in cfDNA are naturally clustered to the clonal hierarchy^1,20^, we defined a mutation cluster as a group of mutations with similar VAFs. The mutation cluster frequency $\theta$ is defined as the fraction of cfDNA carrying the mutations in the cluster, out of all cfDNA mapped to the same genomic positions. Due to the low amount of tumor-derived cfDNA in blood, individual sites may be covered by a very small number of tumor-derived cfDNA reads (or none), leading to highly uncertain estimates of the tumor-derived cfDNA fraction. Therefore, we aggregate tumor-derived signal from multiple sites to obtain a robust estimation. The first step is to identify sites across the genome that are highly likely to be mutated (called potential mutation loci). Specifically, a locus is selected as a potential mutation locus if it meets the following criteria: (a) both matched germline DNA and cfDNA sequencing data have adequate coverage (30 for germline, 80 for cfDNA in this study); (b) bases at the locus in matched germline DNA data contain only reference alleles; (c) the average sequencing error probability is less than the variant’s observed frequency; (d) reads in both matched germline DNA and cfDNA data have high mapping quality (≥20); (e) no strong strand bias is observed; and (f) enough variant supporting reads are observed in the cfDNA data (≥ 3). All potential mutation loci are ranked by read coverage, VAF, and the counts of variant alleles in matched germline DNA data. Next, we estimated $\theta$ by maximizing the likelihood of observing the data at all potential mutation loci ${{\rm{P}}}\left({\boldsymbol{X}}|\theta \right)$, where ${\boldsymbol{X}}=({X}_{1},{X}_{2},\cdots ,{X}_{r},\cdots )$ is the cfDNA sequencing data and ${X}_{r}$ represents all the information (such as sequence and base qualities) contained in a single read $r$. For each locus, we assume that reads are independently sampled from a cfDNA joint-genotype model that is denoted by the triplet ${\boldsymbol{G}}=\left({g}_{T},{g}_{N},{g}_{W}\right)$ where the subscripts $N$, $T$ and $W$ refer to normal cfDNA, tumor-derived cfDNA and WBC DNA respectively. Only the normal cfDNA genotype ${g}_{N}$ and tumor-derived cfDNA genotype ${g}_{T}$ are utilized in this step, because the WBC genotype ${g}_{W}$ is already controlled by potential mutation locus selection (criterion b). All three genotypes are used in (Step 2) and (Step 3), described below. Specifically, ${g}_{W}$ is essential in the later step of the process to remove germline mutations and WBC-derived somatic mutations (clonal hematopoiesis). Based on the independence assumption of reads, the likelihood of $\theta$ at a potential mutation locus is calculated as the product of the probabilities of observing individual reads covering the potential mutation locus, given the parameter $\theta$. We express this relationship as Eq. (1).

1 $$\rm{P}\left({\boldsymbol{X}}|\theta \right)={\prod }_{r\in {R}_{H}}\rm{P}\left({X}_{r}|\theta \right)={\prod }_{r\in {R}_{H}}{\sum }_{{{\boldsymbol{G}}}_{{\boldsymbol{r}}}}\rm{P}\left({X}_{r}|{{\boldsymbol{G}}}_{{\boldsymbol{r}}},\theta \right)\rm{P}\left({{\boldsymbol{G}}}_{{\boldsymbol{r}}}\right)$$

where ${R}_{H}$ is the pool of reads covering selected potential mutation loci and ${{\boldsymbol{G}}}_{{\boldsymbol{r}}}$ is the joint genotype at the potential mutation locus covered by a read $r$. Note that sometimes a read $r$ may cover multiple potential mutation loci, so ${{\boldsymbol{G}}}_{{\boldsymbol{r}}}$ could be the combination of all potential mutation loci covered by read $r$. Since an individual read is sequenced from either tumor-derived cfDNA (with probability $\theta$) or normal cfDNA (with probability $1-\theta$), the likelihood of observing this read can be calculated using a probabilistic mixture model that describes the presence of two subpopulations:

2 $$\rm{P}\left({X}_{r}|{{\boldsymbol{G}}}_{{\boldsymbol{r}}},\theta \right)=\theta \rm{P}\left({X}_{r}|{g}_{{T}_{r}}\right)+\left(1-\theta \right)\rm{P}\left({X}_{r}|{g}_{{N}_{r}}\right)$$

where ${g}_{{T}_{r}}$ and ${g}_{{N}_{r}}$ are the tumor-derived and normal cfDNA genotypes of the potential mutation locus on read $r$. The information contained in an aligned read $r$ (${X}_{r}$) consists of base calls, base qualities and mapping qualities at potential mutation loci in the read. So we can expand ${{\rm{P}}}({X}_{r}|{g}_{{T}_{r}})$ as follows:

3 $$\rm{P}\left({X}_{r}|{g}_{{T}_{r}}\right)=P\left({B}_{r}|{g}_{{T}_{r}}\right)$$

4 $$\rm{P}\left({X}_{r}|{g}_{{N}_{r}}\right)=\rm{P}\left({B}_{r}|{g}_{{N}_{r}}\right)$$

where ${{{B}}}_{{{r}}}$ represents the base call at the potential mutation locus on read *r*. The base quality and the mapping quality are embedded in the probability of sequencing error described below. The probability of error ${\rm{\epsilon }}$ is calculated from the mapping quality ${\rm{m}}$ and the base quality ${\rm{q}}$, as $1-\left(1-{10}^{-\frac{{\rm{m}}}{10}}\right)\left(1-{10}^{-\frac{{\rm{q}}}{10}}\right)$. Assuming that all sequencing error directions have the same probability, the probability of observing a base call given genotype g can be calculated from the probability of error ${\rm{\epsilon }}$. So we have

5 $$	{\rm{P}}({\mathrm{A}}|{\rm{g}})=\left\{\begin{array}{cc}1-\epsilon & {\rm{if}}\,g={\mathrm{AA}}\\ \frac{1}{2}(1-\epsilon )+\frac{1}{6}\epsilon & {\rm{if}}\,g={\mathrm{AB}}\\ \frac{1}{3}\epsilon &{\rm{if}}\,g={\mathrm{BB}}\end{array}\right.$$

where $\mathrm{A}$ and ${\rm{B}}$ are the reference and non-reference alleles respectively. Based on the above formulation, an estimation of the mutation cluster frequency $\theta$ can be achieved by optimizing the likelihood ${{\rm{P}}}\left({\boldsymbol{X}}|\theta \right)$ via the Expectation-Maximization (EM) algorithm or a simple grid search. The output tumor fraction is estimated using the above likelihood model based on the highest VAF cluster of mutations, which is generated from Jenks’ natural break optimization on the VAF of all detected mutations. The probability calculation was performed under python 2.7.14 with preloaded packages numpy^41^, pandas^42^, scipy^43^, and decimal.

(Step 2) Predicting somatic mutation candidates using the joint genotype. After obtaining $\theta$, we can determine the variant status of a genomic position by finding the joint genotype that optimizes the posterior probability of reads at that position. As illustrated in Supplementary Fig. 1(ii), for a given locus, we collected all reads that are aligned to the locus in both cfDNA data and the matched germline DNA data, then computed the posterior probability of each joint genotype from the observed reads. This probability can be modeled by a mixture model similar to that in (Step 1). Subsequently, the joint genotype with the highest posterior probability is adopted as the prediction result at the locus. Somatic mutation candidates are then selected by following the inferred joint genotype. In this step, we used the matched germline data ${{\boldsymbol{X}}}_{{\boldsymbol{W}}}$ from WBC and the cfDNA data ${{\boldsymbol{X}}}_{{\boldsymbol{P}}}$ from plasma cfDNA, consisting of normal cfDNA and tumor-derived cfDNA. For a specific locus, its joint genotype is determined as ${{\boldsymbol{G}}}_{{\boldsymbol{MAP}}}$, the joint genotype that maximizes the posterior probability given the observed data and $\theta$:

6 $${{\boldsymbol{G}}}_{{\boldsymbol{MAP}}}={\arg }{\max }\rm{P}\left({\boldsymbol{G}}|{{\boldsymbol{X}}}_{{\boldsymbol{W}}},{{\boldsymbol{X}}}_{{\boldsymbol{P}}},\theta \right).$$

Using Bayes’ theorem, we have

7 $$\rm{P}\left({\boldsymbol{G}}|{{\boldsymbol{X}}}_{{\boldsymbol{W}}},{{\boldsymbol{X}}}_{{\boldsymbol{P}}},\theta \right)\propto \rm{P}\left({{\boldsymbol{X}}}_{{\boldsymbol{W}}},{{\boldsymbol{X}}}_{{\boldsymbol{P}}}|{\boldsymbol{G}},\theta \right)P\left({\boldsymbol{G}}\right)$$

The probability of observing the data is the product of the probability of observing individual reads. So we have

8 $$\rm{P}\left({{\boldsymbol{X}}}_{{\boldsymbol{W}}},{{\boldsymbol{X}}}_{{\boldsymbol{P}}}|{\boldsymbol{G}},\theta \right)=\rm{P}\left({{\boldsymbol{X}}}_{{\boldsymbol{W}}}|{g}_{W}\right)P\left({{\boldsymbol{X}}}_{{\boldsymbol{P}}}|{g}_{N},{g}_{T},\theta \right),$$

9 $$\rm{P}\left({{\boldsymbol{X}}}_{{\boldsymbol{P}}}|{g}_{N},{g}_{T},\theta \right)={\prod }_{r}{\rm{P}}\left({X}_{r}|{g}_{N},{g}_{T},\theta \right)$$

10 $$\rm{P}\left({{\boldsymbol{X}}}_{{\boldsymbol{W}}}|{g}_{W}\right)={\prod }_{{r}^{{\prime} }}P\left({X}_{{r}^{{\prime} }}|{g}_{W}\right),$$

where ${X}_{r}$ (${X}_{{r}^{{\prime} }}$) stands for a single read $r$ (${r}^{{\prime} }$). In the same way we calculate the likelihood of a given $\theta$, we decompose ${{\rm{P}}}\left({X}_{r}|{g}_{N},{g}_{T},\theta \right)$ and ${{\rm{P}}}\left({X}_{{r}^{{\prime} }}|{g}_{W}\right)$, and get

11 $$\rm{P}\left({\boldsymbol{G}}|{{\boldsymbol{X}}}_{{\boldsymbol{W}}},{{\boldsymbol{X}}}_{{\boldsymbol{P}}},\theta \right)\propto \rm{P}\left({\boldsymbol{G}}\right){\prod }_{r}\left[\left(1-\theta \right)\rm{P}\left({X}_{r}|{g}_{N}\right)+\theta \rm{P}\left({X}_{r}|{g}_{T}\right)\right]{\prod }_{{r}^{{\prime} }}\rm{P}\left({X}_{{r}^{{\prime} }}|{g}_{W}\right)$$

As the majority of normal cfDNA comes from WBCs, we set the prior distribution of the joint genotype $G$ as

12 $$\rm{P}({\boldsymbol{G}}) = \rm{P}({g}_{N},{g}_{T},{g}_{W}) \left\{\begin{array}{cc}\rm{P}({g}_{N},{g}_{T})&{\rm{if}}\;{g}_{W}={g}_{N}, \\ 0&{{\rm{otherwise}}}.\end{array}\right.$$

The joint distribution of the component $({g}_{N},{g}_{T})$ in joint genotype $G$ has been defined in JointSNVMix^44^. It can also be calculated from public databases. Based on the above formulation, the joint genotype can be determined for every locus. By comparing the three components of the joint genotype with the highest posterior probability, we can determine whether the locus is a somatic mutation, a germline mutation, or a loss of heterozygosity (LOH) site. The somatic mutation loci are input as mutation candidates in the next filtration steps. The above model is a probabilistic deconvolution of the normal and tumor signals in cfDNA. By incorporating the matched germline data (WBC) and the mutation cluster frequency $\theta$, we separate the tumor-derived cfDNA from the total cfDNA at individual somatic SNV candidates, and thus enhance mutation detection (as shown in the “Results” section of Experimental analysis of five techniques).

(Step 3) Site-level filtration. To reduce false positives from mutation candidates, we investigated a set of site-level statistics in raw data and FLASh-processed data (i.e., both single-end reads from merged overlapping read pairs and paired-end read pairs without overlapping regions). The site-level statistics used here include averaged base quality, averaged mapping quality, strand bias, depth of coverage, and nearby sequencing context (e.g., repeats and indels). Detailed descriptions and default thresholds for these site-level filters are listed in Supplementary Table 5. One essential filter to determine the mutation candidates in this iterative round is the binomial VAF test. It removes the mutation candidates whose VAF is not likely to be observed based on the current mutation cluster frequency. With the joint-genotype model and the binomial VAF test, the VAF of the mutation candidates in this iteration is around the estimated mutation cluster frequency, and thus these mutation candidates can form a cluster. Based on the results from all filters, each mutation candidate is sorted into one of three categories: pass, hold, or reject. Candidates in the pass category pass all filters, so they are very likely to be mutations. Candidates in the hold category fail some non-essential filters, so we cannot determine whether they are mutations at this step. Candidates in the reject category fail at least one essential filter (e.g. averaged base quality), so they are regarded as false positives and removed from further analysis. The requirements for a variant to be classified as either pass or hold, are listed in Supplementary Table 5.

Iterating (Steps 1–3) to refine the mutation cluster frequency estimate. After (Step 3), we select potential mutation loci from the mutation candidates in the pass category to refine the $\theta$ estimation in (Step 1). By repeating (Steps 1–3) for the same mutation cluster, we obtain a stable frequency estimate and a group of mutation candidates for this cluster. Convergence is reached when the difference between two consecutive $\theta$ estimations is less than 0.01. In our experiments with the 12 plasma samples from the 6 CRPC patients, convergence is usually reached after only two rounds (Supplementary Fig. 11). Thus, with just one iteration of (Steps 1–3), we already accurately capture the most frequent mutation cluster. In fact, our software offers both options: a quick version that performs only one round of estimation and candidate detection for each cluster, and a slow version that iterates until convergence for each mutation cluster.

(Step 4) Output and removal candidates from data. After obtaining somatic mutation candidates from the most frequent mutation cluster, we output the mutation candidates in the pass and hold categories from (Step 3), determining the mutation cluster in this round. Then we remove the loci and data of these sites from the cfDNA data. After removal, we continue iterating from (Step 1) to identify the next most frequent mutation cluster.

Termination criterion. Mutation clusters are detected one at a time, in the decreasing order of their frequency in cfDNA. The process terminates until no mutation candidates are found in (Step 4) (i.e., the pass and hold categories are empty).

(iii) Error filtration at the read level. Site-level statistics provide some information on the difference between sequencing errors and true mutations, but are not adequate for error filtration in cfDNA. Due to the low tumor fraction and high heterogeneity of cfDNA, site-level frequency estimates are uncertain and unreliable for mutations with only a few supporting reads. To reduce the number of false positives among mutation candidates, we developed a machine learning filter to eliminate reads with sequencing errors at candidate sites and remove SNV candidates whose count of confirmed supporting reads fails to pass a threshold (see details in Supplementary Table 6). Specifically, for each mutation candidate, we classify each of its supporting reads with a random forest model in order to distinguish sequencing errors from true variants. This model combines a variety of features (Supplementary Table 7) and automatically discovers statistical relationships among the features that reflect sequencing errors. It is worth noting that read pair statistics (e.g., fragment length and features of the read mate) are always among the most informative features of the random forest model. Since this error filtration method is applied at the read level, it improves the precision of detecting low-frequency somatic mutations. Although this read-level filter can be performed at any step of the method (e.g., after alignment or during the iterations), we prefer to perform it at the end of the cfSNV workflow in order to save computing time and resources. Generally, the later this step is performed, the fewer sequencing reads need to be inspected for errors, and thus less time is needed for cfSNV. Practically, based on our hands-on experience of the real data, the time needed to inspect read-level errors in the beginning of the process is reduced 50 fold if it is performed at the end of the process: that is, for each read that needs to be inspected at the end of the process, at least 50 reads would have needed to be inspected at the beginning.

To train the random forest model, we used four WES sequencing datasets from the same cancer patient (MBC_315): two cfDNA sequencing datasets, a WBC sequencing dataset, and a tumor biopsy sequencing dataset. As the two cfDNA sequencing datasets were obtained from the same cfDNA sample, we can treat them as technical replicates and label their read pairs by their concordance. The training data are the supporting cfDNA read pairs at known mutation/error sites and are labeled as containing mutations or errors. Mutation sites are defined as the collection of common germline mutations detected using Strelka2 germline^45^ from all four datasets. In addition, common somatic mutations were detected using Strelka2 somatic and MuTect^16^ from two cfDNA-WBC pairs (cfDNA data vs. WBC data) and one tumor-WBC pair (tumor data vs. WBC data). Error sites are defined as sufficiently covered sites (> 80x) with only one high-quality non-reference read (base quality ≥20 and mapping quality ≥40) in all four datasets. All labeled read pairs were extracted from raw cfDNA data using picard tools FilterSamReads (Supplementary Table 8). Different features were extracted from the overlapping read pairs and the non-overlapping read pairs (Supplementary Table 7). Genome sequences around the mutation candidates were extracted from hg19 using bedtools^46^. All categorical features were expanded using one-hot encoding method. We used the parameters of the random forest model as follows: (1) the number of decision trees is 100, (2) the maximum tree depth is 10, (3) imbalanced classes were handled by setting the class weights with option balanced, and (4) other parameters were left at their default values. Two random forest classifiers (for overlapping read pairs and non-overlapping read pairs) were trained on read pairs extracted from the WES data (SRR6708941) using RandomForestClassifier from the python library scikit-learn^47^. Read pairs from SRR6708920 were only used for validating the model. The trained classifiers are saved in the cfSNV code package (see "Code Availability").

### Truncal-bTMB measure

Somatic SNVs are annotated using snpEff^48^. Nonsynonymous mutations and high-impact mutations are treated the same in snpEff results. Mutations from Strelka2 were filtered if their VAF in the matched normal is greater than 1%. As the mutation’s VAF in cfDNA reflects the clonality of a mutation, we treat a mutation as a truncal mutation if its VAF is greater than a threshold; otherwise it is a branch mutation. The threshold is defined as 60% of the average VAF of the 5 most frequent mutations. The truncal-bTMB measure can then be calculated as the sum of the normalized VAFs of all truncal nonsynonymous mutations.

13 $${\mathrm{truncal}}{\mbox{-}}{\mathrm{bTMB}}=\frac{\sum ({\rm{VAF}}\;{\rm{of}}\;{\rm{truncal}}\;{\rm{mutations}})}{\sum ({\mathrm{highest}}\;5\;{\mathrm{VAF}})/5}$$

### Additional validation data for random forest classifier

To further test the random forest classifiers, we generated data from other patients with metastatic breast or prostate cancer (Supplementary Table 9). For each patient, we obtained WES data of a WBC sample, a tumor biopsy sample, and plasma samples from two different time points. To generate the testing data and label the individual reads, we used the same procedure as described in the “Methods” section of Error filtration at the read level for producing the training data.

### Simulation with BAMSurgeon to evaluate precision and sensitivity

To evaluate the performance of cfSNV, we collect cfDNA samples and WBC samples from three healthy individuals. We generated two WES technical-replicate datasets (denoted as X and Y) from the cfDNA sample and one WES dataset (denoted as W) from the genomic DNA of the matched WBC sample of each individual (see Supplementary Fig. 2). We then simulated the WES data of six virtual cancer patients from the data of these three healthy individuals. Specifically, given the two cfDNA WES datasets from one healthy person (X and Y), we inserted mutations at different VAFs (generated by BAMSurgeon^49^) into X (or Y) to simulate the data of a virtual cancer patient’s cfDNA sample (denoted as X_mutated_ (or Y_mutated_)), while we treated W as the data of the simulated WBC sample of the same virtual cancer patient (see Supplementary Fig. 2). Therefore, this strategy used two cfDNA datasets and one WBC dataset from one healthy person to simulate the data of two virtual cancer patients. Specifically, for simulating each cfDNA sample (i.e., X_mutated_ and Y_mutated_), the BAMSurgeon program inserted 3000 in sillico somatic SNVs with different VAFs: 300 at 15%, 300 at 13%, 300 at 10%, 300 at 8%, 300 at 5%,300 at 3%, 600 at 1%, and 600 at 0.5%. A total of 9912 mutations were successfully inserted into the WES data of six cfDNA replicates from three healthy individuals. These successfully inserted mutations were regarded as the ground-truth somatic SNVs. There were some mutations that failed to be inserted into the sequencing data, because their VAFs were incompatible with the sequencing depth in the original data. For example, a mutation with VAF 1% cannot exist in the data with a sequencing depth of 50 reads. Since BAMSurgeon treats read pairs as single reads, we disabled the features from overlapping read mates in the read-level filtration model. In other words, all read pairs were treated as non-overlapping read pairs in the read-level filtration step. We evaluated the performance of cfSNV, MuTect (disabling the contamination filters and tumor_lod = 5.25) and Strelka2 (default parameters, with enabled and disabled filters) on this simulation dataset by comparing the SNVs identified from each algorithm with the ground-truth somatic SNVs (i.e., the inserted in sillico somatic SNVs). For a fair comparison, we tried all combinations of the main parameter settings for cfSNV, MuTect, and Strelka2, such that they all had similar specificities, i.e., false positive rates ranging from 1.07 to 1.15/Mb, and then we compared sensitivity at this specificity.

We also evaluated the performance of cfSNV on a higher coverage simulated dataset (see Supplementary Table 1). For this dataset, the input to BAMSurgeon was a pool of cfDNA data from eight cancer patients (MBC_333, MBC_336, MBC_292, CRPC_531, MBC_284, CRPC_525, MBC_303, and MBC_335)^21^. Before mixing the eight cfDNA samples, to avoid the potential interference of the germline and somatic mutations in the individual cfDNA samples, we removed the reads covering these positions. The germline mutations were identified using a standard pipeline (GATK HaplotypeCaller) from individual samples; the somatic mutations were identified using cfSNV, MuTect, and Strelka2 from the individual cfDNA samples and their matched WBC samples. Three methods were used in the somatic mutation removal to avoid potential bias introduced in this step. Sequencing reads in the individual data were removed if they fell in a 200 bp region centered at any germline/somatic mutations (upstream 100 bp and downstream 100 bp). Then the eight individual cfDNA samples were merged. The mean target coverage of the pooled sample reached 2200×. The BAMSurgeon program attempted to insert 1000 somatic SNVs with different VAFs: 100 at 8%, 100 at 5%, 100 at 3%, 100 at 1%, 100 at 0.8%,100 at 0.5%, 200 at 0.3%, and 200 at 0.1%. A total of 581 mutations were successfully inserted. The inserted mutations were regarded as the ground-truth somatic SNVs.

### Mutation concordance between tumor biopsy and plasma samples

To validate our method on real data, we examined mutation concordance between a tumor biopsy sample and the plasma samples. This analysis involves twelve patients with metastatic breast cancer and six patients with metastatic prostate cancer^21^. Each patient had a tumor biopsy sample, a WBC sample, and plasma samples from two different time points, all processed with WES. Mutations called from one plasma sample were checked in the raw sequencing data of the matched tumor biopsy sample and the other plasma sample. A somatic SNV is confirmed if there are at least three reads supporting the variant allele in the matched tumor biopsy sample or at least three reads supporting it in the other plasma sample. A somatic SNV is not confirmed when the mutation has power at least 0.9 and fewer than 3 alternative reads^21^.

### Comparison with MuTect and Strelka2 on real cfDNA data

We compared our method to two state-of-the-art methods, Mutect and Strelka2. The same validation analysis was conducted for both methods on the same samples. Both tools were run with their default parameters unless otherwise noted in the text. The same confirmation process described in the “Methods” section of Mutation concordance between tumor biopsy and plasma samples was conducted for somatic SNVs detected by MuTect and Strelka2.

### Calculation of TMB and bTMB

For tissue biopsy samples, we called their somatic SNVs using Strelka2. The mutations were annotated using snpEff. TMB was calculated as the number of nonsynonymous SNVs. For plasma samples, we called somatic mutations using MuTect, Strelka2 or cfSNV, and annotated them using snpEff. Mutations from Strelka2 were filtered if their VAF in the matched normal is greater than 1%. We calculated traditional bTMB as the count of all nonsynonymous mutations with VAF ≥ 0.15.

### Simulation with BAMSurgeon to evaluate the accuracy of the intelligent search for the most frequent mutation cluster

We used BAMSurgeon to generate simulation data. The input to BAMSurgeon was the WBC sequencing data from MBC_299. The program attempted to insert 300 mutations at three different VAF levels: 50 mutations at 20%, 150 mutations at 8%, and 100 mutations at 2%. Five simulated samples with the same settings were generated.

### Generating spike-in simulation data to validate the mutation cluster frequency estimates

To evaluate the accuracy of our mutation cluster frequency estimation, we generated spike-in simulation data by mixing the primary tumor sequencing data (ERS700859) and the WBC sequencing data (ERS700858) of a metastatic breast cancer patient, at varying concentrations of cfDNA reads (from 2 to 20% in eight steps). Five independent mixtures are generated at every concentration. Each spike-in sample contains a total number of randomly sampled reads equivalent to 170x coverage of the targeted regions. The coverage of the targeted regions is limited by the number of sequencing reads in the original data.

### Impact of the mutation cluster frequency on the model-to-data fitness at a single simulated mutation

The model-to-data fitness is evaluated using the likelihood ratio ${L}_{\theta }$, the ratio between the maximum likelihood of a somatic-mutation joint genotype (i.e., homozygous and heterozygous genotypes) and the maximum likelihood of a non-somatic-mutation joint genotype (other joint genotypes) given $\theta$. Since we screened mutation candidates based on the joint genotype estimated at each position, this likelihood ratio reflects the ability of cfSNV to detect a somatic mutation candidate. We explored the theoretical properties of this likelihood ratio using simulated mutations, which consist of randomly generated base quality values, mapping quality values and a corresponding list of base calls reflecting the VAF. To compare the fitness of the model with and without $\theta$, we calculated the value of ${L}_{\theta }/{L}_{1}$.

### Impact of the mutation cluster frequency on real patient data

To test the impact of estimated mutation cluster frequency on real patient data, we selected four samples whose frequent mutation clusters have low frequency <20% estimated from cfSNV and ichorCNA. We performed cfSNV on the four samples using both a predetermined value of $\theta$ (0.2, 0.5, 0.8, and 1.0) and the estimated $\theta$ of the most frequent mutation cluster in the sample. When we set $\theta$ as 1.0, the candidate screening model is the same as the regular joint genotype model for solid tumor samples, which is equivalent to a model that does not incorporate the estimated mutation cluster frequency. In this simulation, we also disabled the iterative procedure to converge on the best value of $\theta$, so the candidate screening only took place at the given $\theta$.

### Rescuing mutations from conventional post-filtration

The clustered read position, defined as a position where the alternative alleles are clustered at a constant distance from the start and end of the read alignment^16^, is regarded as a hallmark of misalignment artifacts. Because of the existence of the preferred start and end positions, the start and end sites of reads at some mutations tend to cluster together, and thus the position of the alternative alleles on these reads tend to cluster together. Therefore, cfDNA preferred start and end positions may make the true somatic mutations look like misalignment false positives with clustered read position. To rescue these mutations, we removed the conventional clustered read position filter entirely. Instead, to remove misalignment artifacts, we implemented a filter that simultaneously checks the co-occurrence of candidates and mismatch positions on the reads with alternative alleles (variant supporting reads), instead of purely relying on the clustered read position of a single mutation. If multiple candidates and mismatch positions exclusively co-occur on the variant supporting reads, we regard them as artifacts from misalignment (Supplementary Table 5). A mutation is called rescued if it is detected by cfSNV but would be filtered by conventional methods due to the clustered read position. For each rescued mutation, the same confirmation process described in the “Methods” section of Mutation concordance between tumor biopsy and plasma samples was conducted. The fraction of confirmed rescued mutations among all rescued mutations was calculated for every sample. Indeed, we were able to confirm that for some rescued mutations, the variant bases are more clustered in cfDNA reads than in solid tumor samples (Supplementary Fig. 12), validating our rationale.

### Reporting summary

Further information on research design is available in the Nature Research Reporting Summary linked to this article.

## Supplementary information

Supplementary Information

Reporting Summary

## Source data

Source data

## Data Availability

Raw sequencing data that support the findings of this study have been deposited into European Genome-Phenome Archive under accession code EGAS00001004373 (https://ega-archive.org/datasets/EGAD00001006096). The data is available under restricted access, which can be obtained by contacting the corresponding author. The public data used in this study are following: the public WES data from the 12 MBC patients and the 6 CRPC patients^21^ are available in dbGaP under the accession code phs001417.v1.p1; the public WES data from the breast cancer patient with liver metastasis^22^ are available in European Nucleotide Archive under the accession numbers ERS700858, ERS700859, ERS700860, and ERS700861. The remaining data are available within the Article, Supplementary Information, or Source data. Data under phs001417.v1.p1 are used for the validation of cfSNV and are associated with Figs. 2–4, Supplementary Figs. 3–8, 11, 12, and Supplementary Table 1–4, 8, 9. Data under ERS700858, ERS700859, ERS700860, and ERS700861 are used for the validation of cfSNV and are associated with Fig. 4. Data under EGAS00001004373 are used for the application on NSCLC patients and the validation of cfSNV, and are associated with Figs. 2, 5, Supplementary Figs. 6, 9, 10, and Supplementary Table 4. Source data are provided with this paper. For information on the use for a commercial purpose or by a commercial or for-profit entity, please contact Prof. Xianghong Jasmine Zhou (https://zhoulab.dgsom.ucla.edu/) to obtain a materials transfer agreement.

## Code availability

cfSNV can be obtained https://github.com/jasminezhoulab/cfSNV. It can be freely used for educational and research purposes by non-profit institutions and U.S. government agencies only under the BSD license. For information on the use for a commercial purpose or by a commercial or for-profit entity, please contact Prof. Xianghong Jasmine Zhou (https://zhoulab.dgsom.ucla.edu/).
