# Author Correction: Sensitive detection of tumor mutations from blood and its application to immunotherapy prognosis

**DOI:** 10.1038/s41467-026-69268-5

**Published:** 2026-02-05

**Authors:** Shuo Li, Zorawar S. Noor, Weihua Zeng, Mary L. Stackpole, Xiaohui Ni, Yonggang Zhou, Zuyang Yuan, Wing Hung Wong, Vatche G. Agopian, Steven M. Dubinett, Frank Alber, Wenyuan Li, Edward B. Garon, Xianghong Jasmine Zhou

**Affiliations:** 1 Department of Pathology and Laboratory Medicine, David Geffen School of Medicine, University of California at Los Angeles, Los Angeles, CA USA; 2 Bioinformatics Interdepartmental Graduate Program, University of California at Los Angeles, Los Angeles, CA USA; 3 Institute for Quantitative & Computational Biosciences, University of California at Los Angeles, Los Angeles, CA USA; 4 Department of Medicine, David Geffen School of Medicine at UCLA, Los Angeles, CA USA; 5 EarlyDiagnostics Inc, Los Angeles, CA USA; 6 Department of Statistics, Stanford University, Stanford, CA USA; 7 Department of Biomedical Data Science, Stanford University, Stanford, CA USA; 8 Department of Surgery, David Geffen School of Medicine at UCLA, Los Angeles, CA USA; 9 Department of Molecular and Medical Pharmacology, David Geffen School of Medicine at UCLA, Los Angeles, CA USA; 10 VA Greater Los Angeles Health Care System, Los Angeles, CA USA; 11 Department of Microbiology, Immunology and Molecular Genetics, University of California at Los Angeles, Los Angeles, CA USA

Correction to: *Nature Communications* 10.1038/s41467-021-24457-2, published online 7 July 2021

In the version of the article initially published, the Competing interests section was incomplete and has now been expanded in the HTML and PDF versions of the article to: “X.J.Z., W.L., and W.H.W. are co-founders of EarlyDiagnostics. X.J.Z and W.H.W serve on the Board of Directors for EarlyDiagnostics. X.J.Z. has an executive leadership position at EarlyDiagnostics. S.M.D. is a scientific advisor to EarlyDiagnostics. X.N., S.L., and M.L.S. are employees of EarlyDiagnostics. S.L., W.Z., M.L.S., X.N., Y.Z., S.M.D. have stock options with EarlyDiagnostics. X.J.Z., W.L., W.H.W., and F.A. are stockholders of EarlyDiagnostics. W.Z., Y.G., and W.L. are consultants for EarlyDiagnostics. X.J.Z., S.L. and W.L. are inventors on a patent application submitted by the Regents of the University of California and licensed to EarlyDiagnostics (Patent No. US20210125683A1). The other authors have no competing interests to declare.”

