# Supplementary material for: Sensitive detection of tumor mutations from blood and its application to immunotherapy prognosis
Source: Nat Commun. 2021 Jul 7;12:4172. doi: 10.1038/s41467-021-24457-2 (PMC8263778; doi:10.1038/s41467-021-24457-2)
Supplement: Supplementary file 1 — Supplementary Information [file 41467_2021_24457_MOESM1_ESM.pdf]

Supplementary Figures

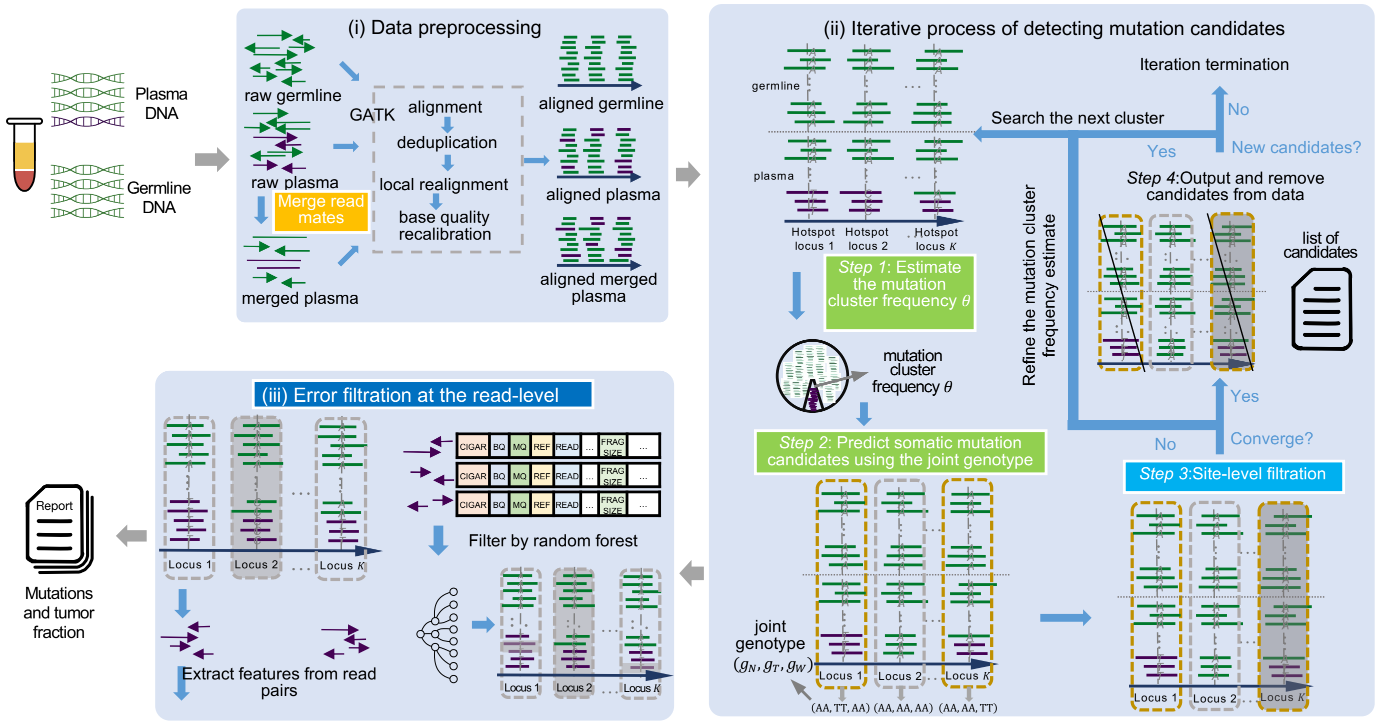

Supplementary Fig. 1 | Workflow of cfSNV. cfSNV takes plasma DNA and germline DNA sequencing data as inputs. It first merges overlapping read mates in cfDNA sequencing data. The reads are processed using the GATK pipeline. After these steps, an iterative procedure estimates the mutation cluster frequency θ based on a set of carefully selected hotspots. Each iteration step determines the joint tumor-normal genotypes across sequencing regions, then eliminates somatic SNV candidates that fail essential filters based on site-level statistics (Methods). Mutation candidates are used as hotspot sites to refine θ and candidate detection until the frequency converges. SNV candidates from the previous iteration are output and masked before the next iteration. After all candidates are detected, a random forest classifier identifies raw read pairs with sequencing errors. Finally, somatic SNVs are reported only if enough variant supporting read pairs passed the random forest screening.

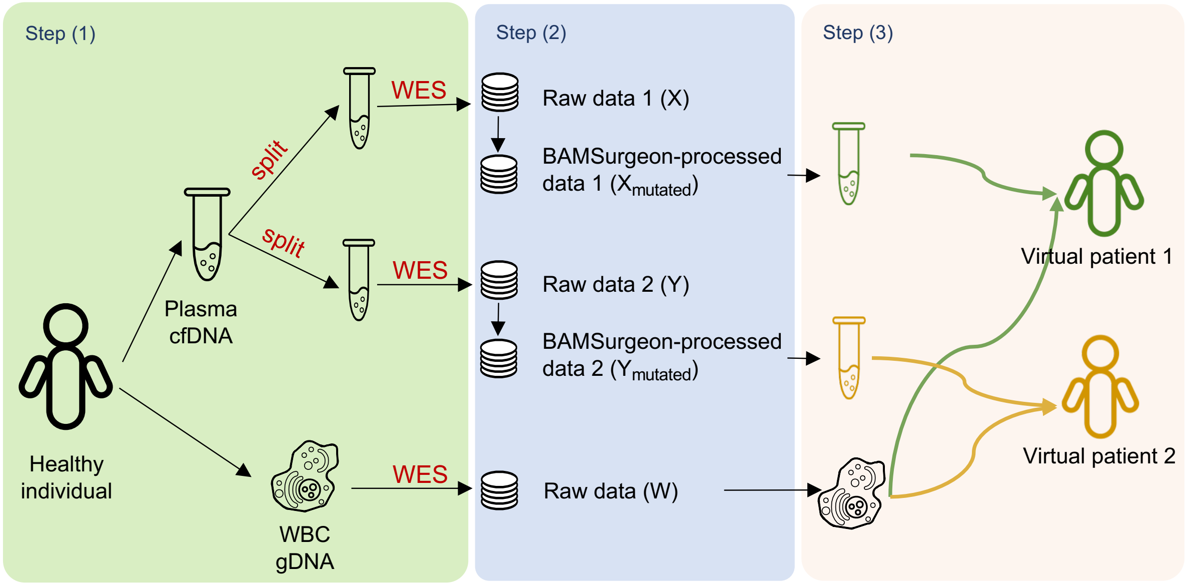

Supplementary Fig. 2 | Generation of the simulation data from cfDNA samples and WBC samples of three healthy individuals. We used the plasma samples and the WBC samples of three healthy individuals to simulate the WES data of the cfDNA sample and the matched WBC sample of six

virtual cancer patients. Specifically, for samples of each healthy individual, we performed a three-step simulation procedure (see Methods) to generate the simulation data of two virtual cancer patients: (1) we split this healthy people's plasma cfDNA into two equal-sized portions (called two technical replicates), then each portion was used to independently generate the real WES data (with sequencing depth ~200x). This procedure resulted in two different WES datasets, raw data 1 (X) and raw data 2 (Y), which carry no mutations. We also generated one WES dataset, raw data (W), from the genomic DNA of the matched WBC sample of the healthy individual. (2) A set of predefined in silico somatic SNVs were independent generated by BAMSurgeon and were added into X, resulting in a WES dataset BAMSurgeon-processed data 1 ($X_{mutated}$), which carry mutations; analogously, from Y the BAMSurgeon-processed data 2 ($Y_{mutated}$) was generated. (3) $X_{mutated}$ and W simulated the WES data of the cfDNA and the matched germline DNA of a virtual cancer patient; and $Y_{mutated}$ and W simulated the two WES datasets of another virtual cancer patient. Therefore, in this simulation procedure, two cfDNA WES datasets and one WBC dataset of one healthy individual can be used to generate two virtual cancer patients' data. By applying this simulation procedure to plasma samples of three healthy individuals, we simulated WES data of six virtual cancer patients, where each virtual patient has both the cfDNA data and the matched germline DNA data.

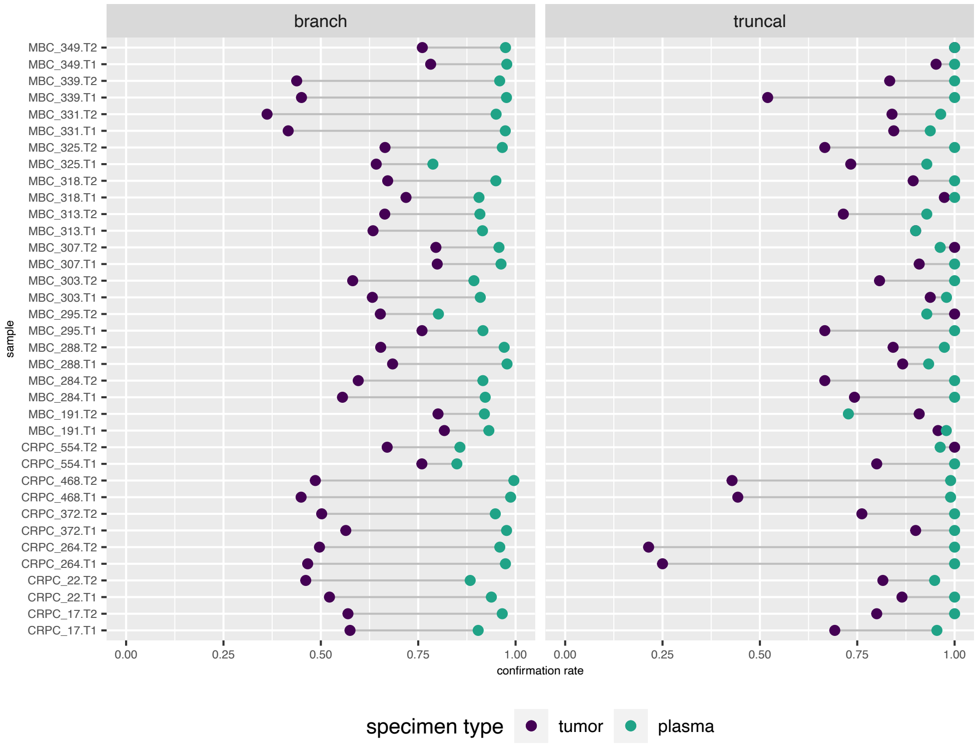

Supplementary Fig. 3 | Fraction of confirmed truncal mutations and branch mutations detected by cfSNV on patient data (n = 36). Mutations found in cfDNA sequencing data were validated by variant supporting read counts, either in cfDNA sequencing data from the other plasma sample or in genomic DNA sequencing data from a tumor biopsy sample collected from the same patient. The clonality of mutations was determined by their relative VAFs.

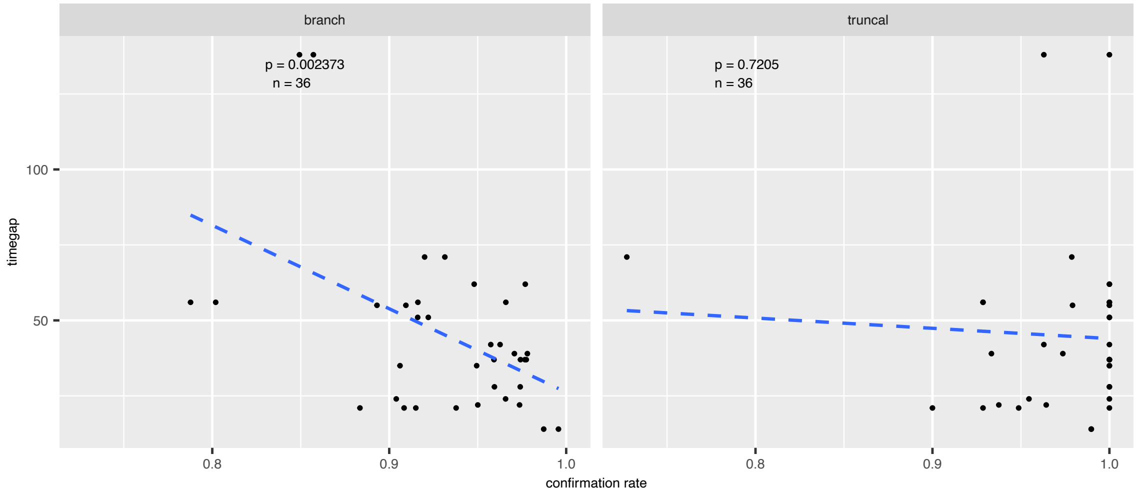

Supplementary Fig. 4 | Correlation on the truncal and branch mutation confirmation rates in the 36 plasma samples with respect to the sample collecting time gap. The p-values were calculated from two-sided Pearson's correlation test. The Pearson's correlation is -0.49 (95% confidence interval

(CI) = [-0.71, -0.19], two-sided Pearson's correlation test p = 0.002373, t statistic = -3.28, degree of freedom (df) = 34) and -0.06 (95% CI = [-0.38, 0.27], two-sided Pearson's correlation test p = 0.7205, t statistic = -0.36, df = 34) for branch mutations and truncal mutations respectively.

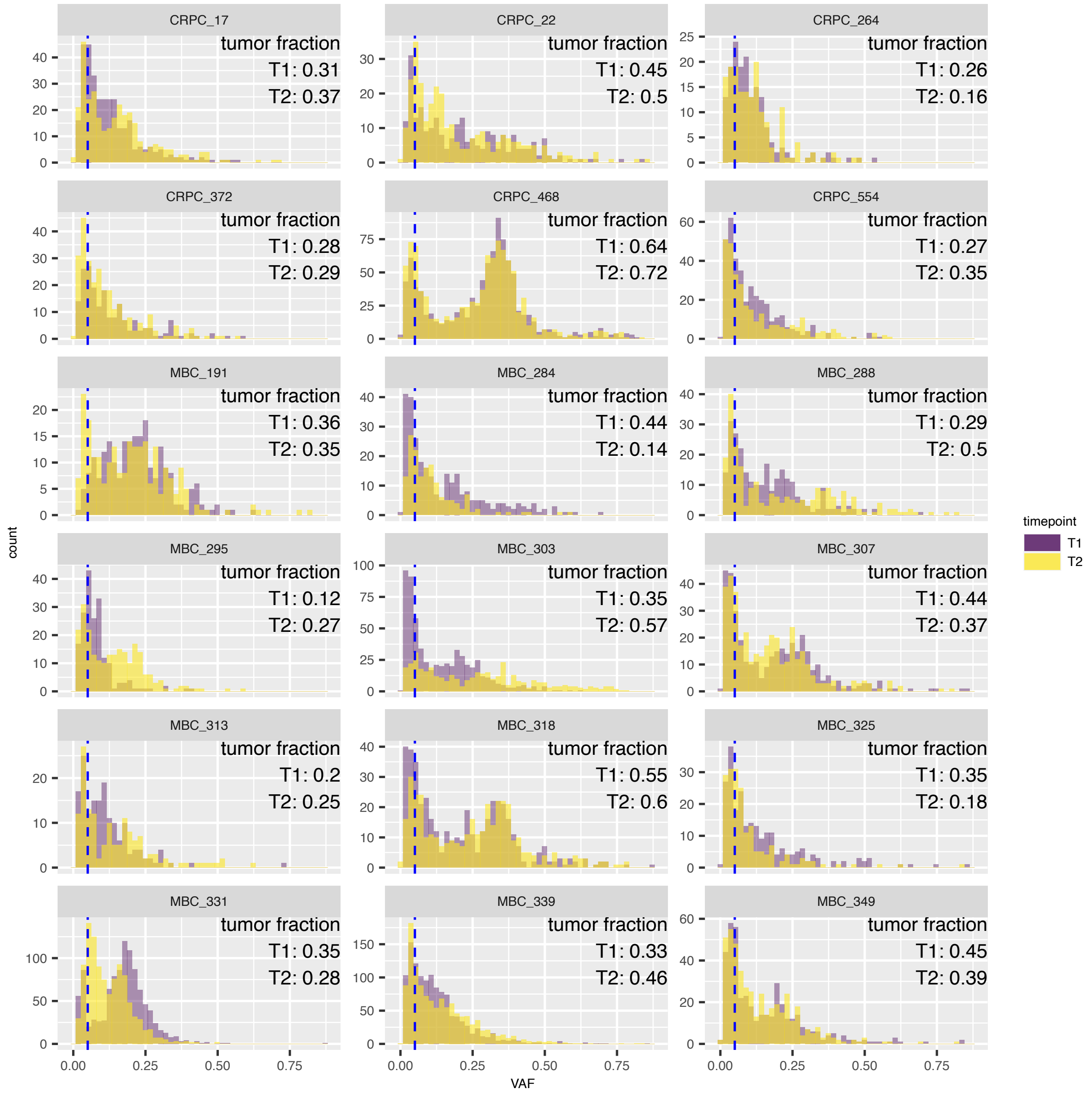

Supplementary Fig. 5 | Histograms of variant allele frequency of somatic mutations detected by cfSNV in each sample in the validation patient cfDNA data (n = 36). T1 refers to the plasma sample collected from the first time point; T2 refers to the plasma sample collected from the second time point.

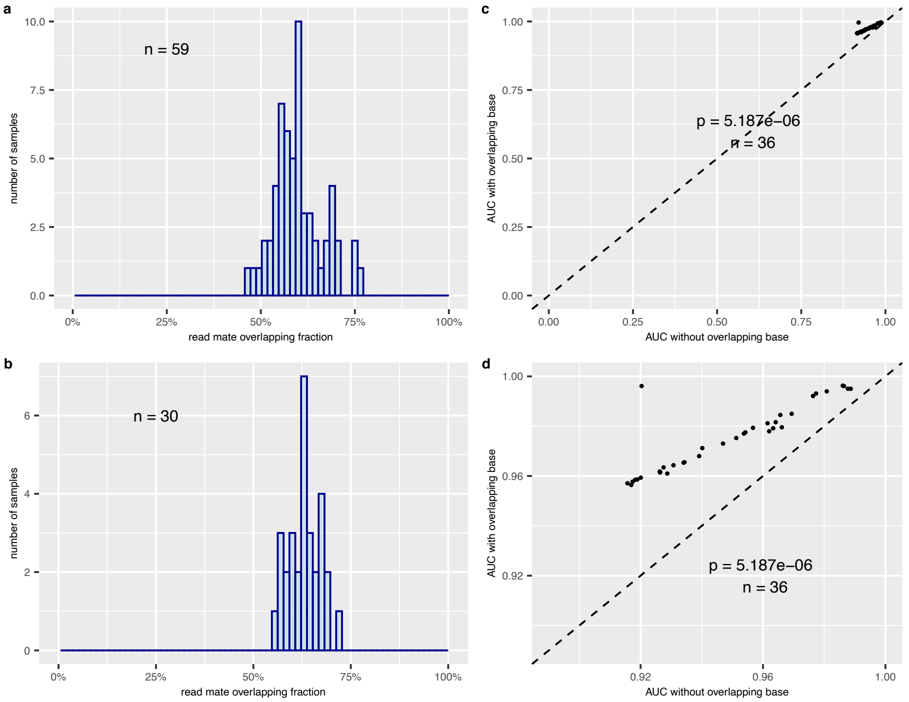

Supplementary Fig. 6 | Quantifying the existence and impact of overlapping read mates in cfDNA sequencing data. a, Histogram of the fraction of merged overlapping read mates in 59 cfDNA whole exome sequencing samples from metastatic cancer patients (paired-end 2x100bp). **b,** Histogram of the fraction of merged overlapping read mates in 30 cfDNA whole exome sequencing samples from NSCLC patients (paired-end 2x150bp). **c,** Comparison of AUC metrics from classifiers trained on overlapping read pairs and non-overlapping read pairs on 36 testing samples. **d,**A zoom-in figure of Supplementary Fig. 6c. The p-values in **c** and **d** were calculated from one-sided Wilcoxon rank sum test, where the corresponding W statistic = 256.

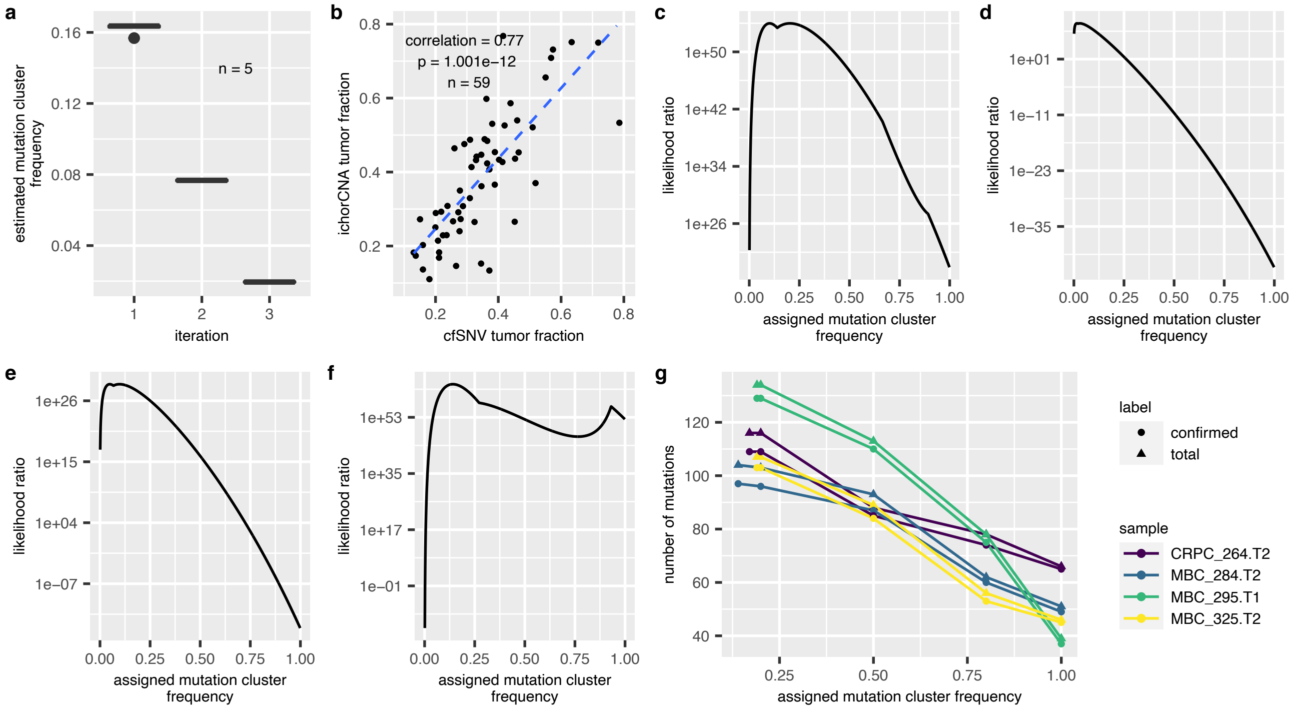

Supplementary Fig. 7 | Simulation on the estimated mutation cluster frequency. **a,** Performance of mutation cluster frequency estimation in the first experiment with simulated samples containing purely synthetic mutations inserted at known VAF levels (20%, 8% and 2%). Each box in this plot shows the estimated mutation cluster frequency for the synthetic mutation cluster at the same VAF level in independent simulation samples (n = 5). In iteration 1, the minima, maxima, center, upper quantile, and lower quantile are 0.157, 0.165, 0.164, 0.164, and 0.163, respectively; in iteration 2, the minima, maxima, center, upper quantile, and lower quantile are 0.076, 0.077, 0.077, 0.077, and 0.076, respectively; in iteration 3, the minima, maxima, center, upper quantile,

and lower quantile are 0.019, 0.020, 0.019, 0.019, and 0.020. **b,** Performance of mutation cluster frequency estimation in the third experiment with cfDNA data (n = 59). The graph demonstrates the Pearson's correlation = 0.77 (95% CI = [0.64, 0.86]) between the tumor fractions estimated by cfSNV and ichorCNA on different sequencing experiments using the same cfDNA samples. The p-value was calculated from the two-sided Pearson's correlation test, p = 1.001e-12, t statistic = 9.1154, df = 57. **c-f,** The likelihood ratio plot of a simulated mutation with VAF 0.1 (**c**), 0.01 (**d**), 0.05 (**e**), and 0.2 (**f**), under varied mutation cluster frequencies. **g,** The number of confirmed mutations and all mutations detected using different mutation cluster frequencies on four plasma samples whose significant mutation clusters have prevalence ≤ 20%. The left most point on each line showed the number of mutations detected at the estimated mutation cluster frequency.

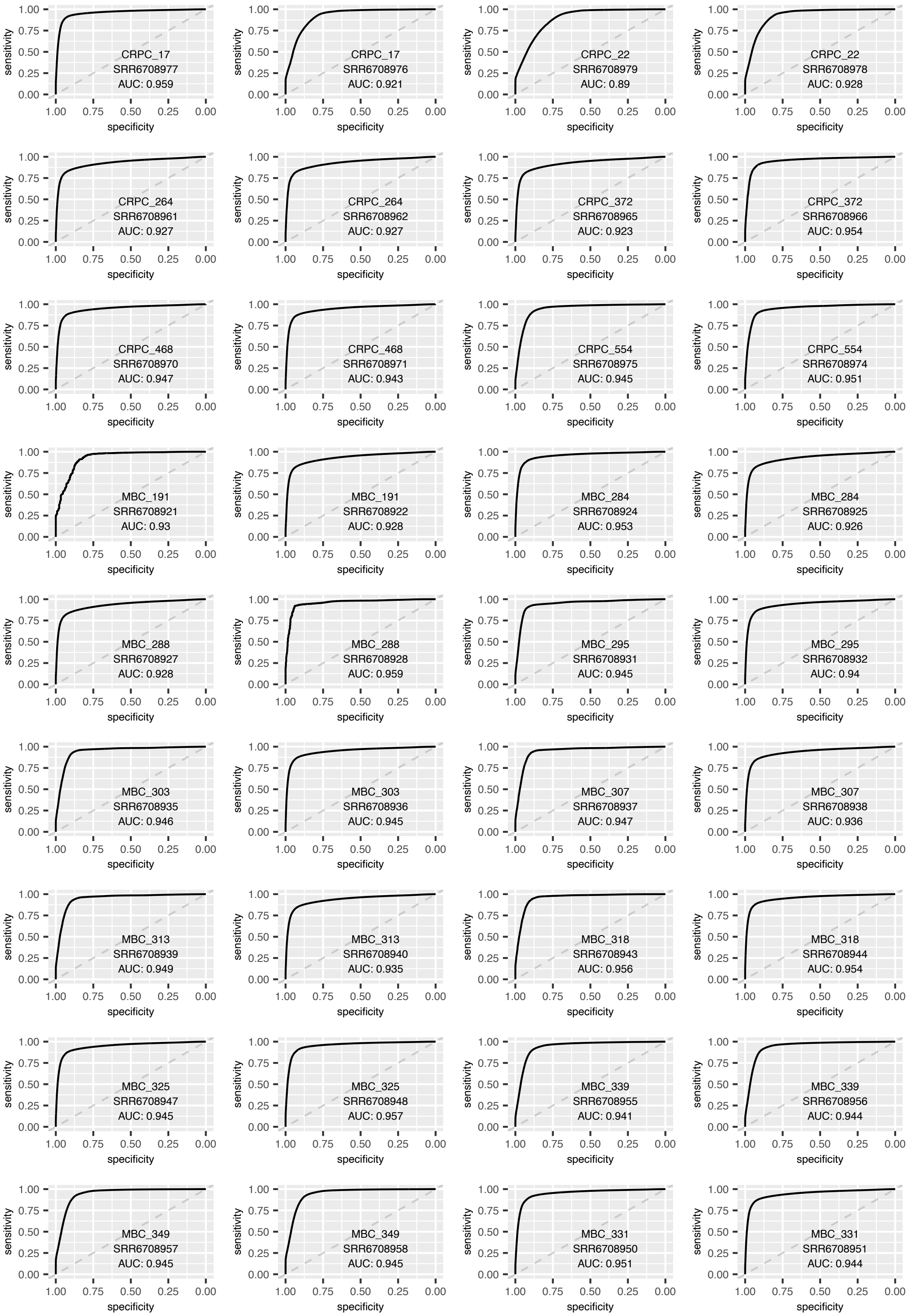

Supplementary Fig. 8 | ROC curves for random forest classifiers on all read pairs in out-of-sample tests. The classifiers were trained using data derived from only WES data of cfDNA sample from a single patient (patient MBC_315, sample SRR6708941). Each independent testing dataset (from one patient) has its own ROC curve. The numbers in parentheses are area under curve (AUC) metrics. The x-axis is false positive rate (FPR); the y-axis is true positive rate (TPR).

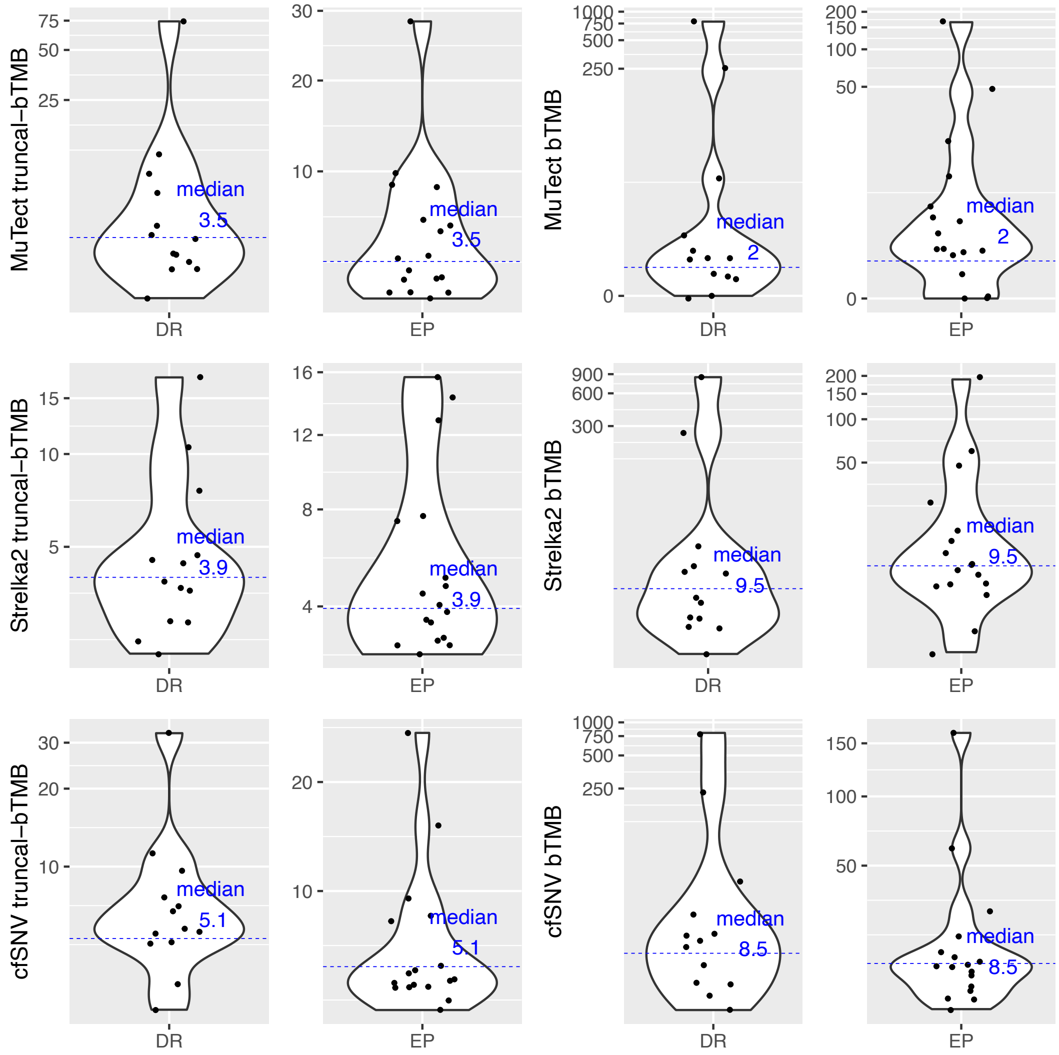

Supplementary Fig. 9 | Distribution of bTMB and truncal-bTMB in the 30 NSCLC patients. The durable responders (DR, PFS > 9 months) and early progressors (EP, PFS < 6 months) are defined based on the outcome of the patients, i.e. progression-free survival. The cutoff for the two patient groups based on bTMB and truncal-bTMB is marked as the dashed blue line.

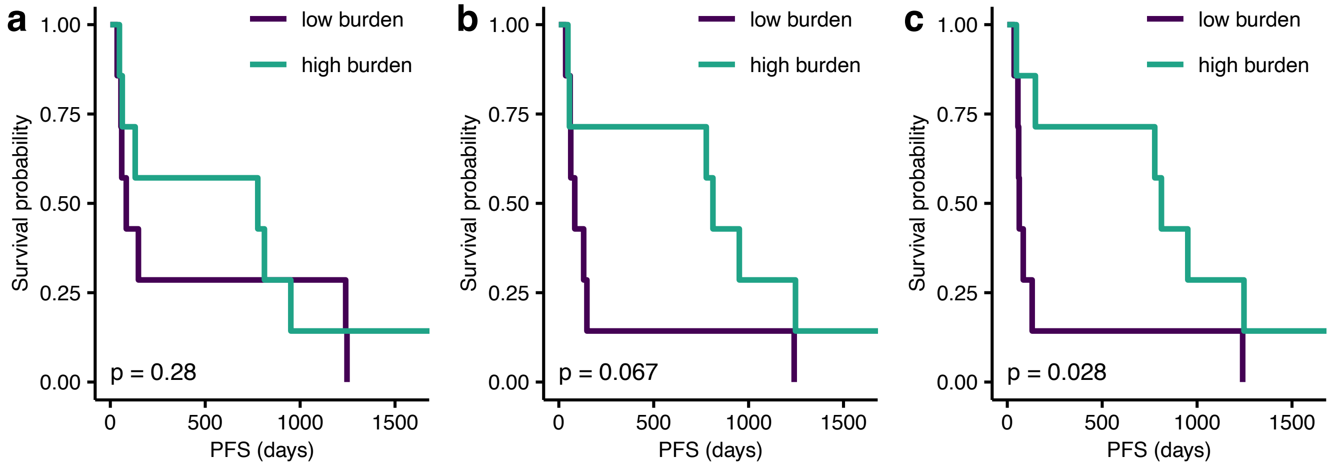

Supplementary Fig. 10 | Kaplan-Meier curves for progression-free survival (PFS) on 14 advanced non-small cell lung cancer patients. a-c, PFS for 14 patients with both tumor biopsy and pre-treatment cfDNA sequencing data. The high-burden and low-burden groups in each plot are defined by the median value of the measure: TMB (**a**, HR=0.721, 95% CI [0.239, 2.173], Z statistic = -0.61), bTMB (**b**, HR=0.411, 95% CI [0.124, 1.355], Z statistic = -1.43), or truncal-bTMB (**c**, HR=0.326, 95% CI [0.098, 1.079], Z statistic = -1.77). All p-values were calculated from one-sided log-rank test.

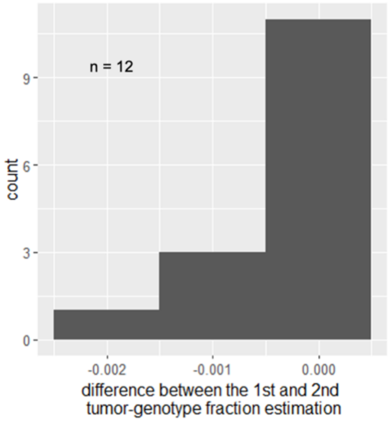

Supplementary Fig. 11 | Histogram of the difference between the tumor-genotype fractions estimated in the first round and the second round (refined by mutation candidates) for the 12 plasma samples of the 6 CRPC patients.

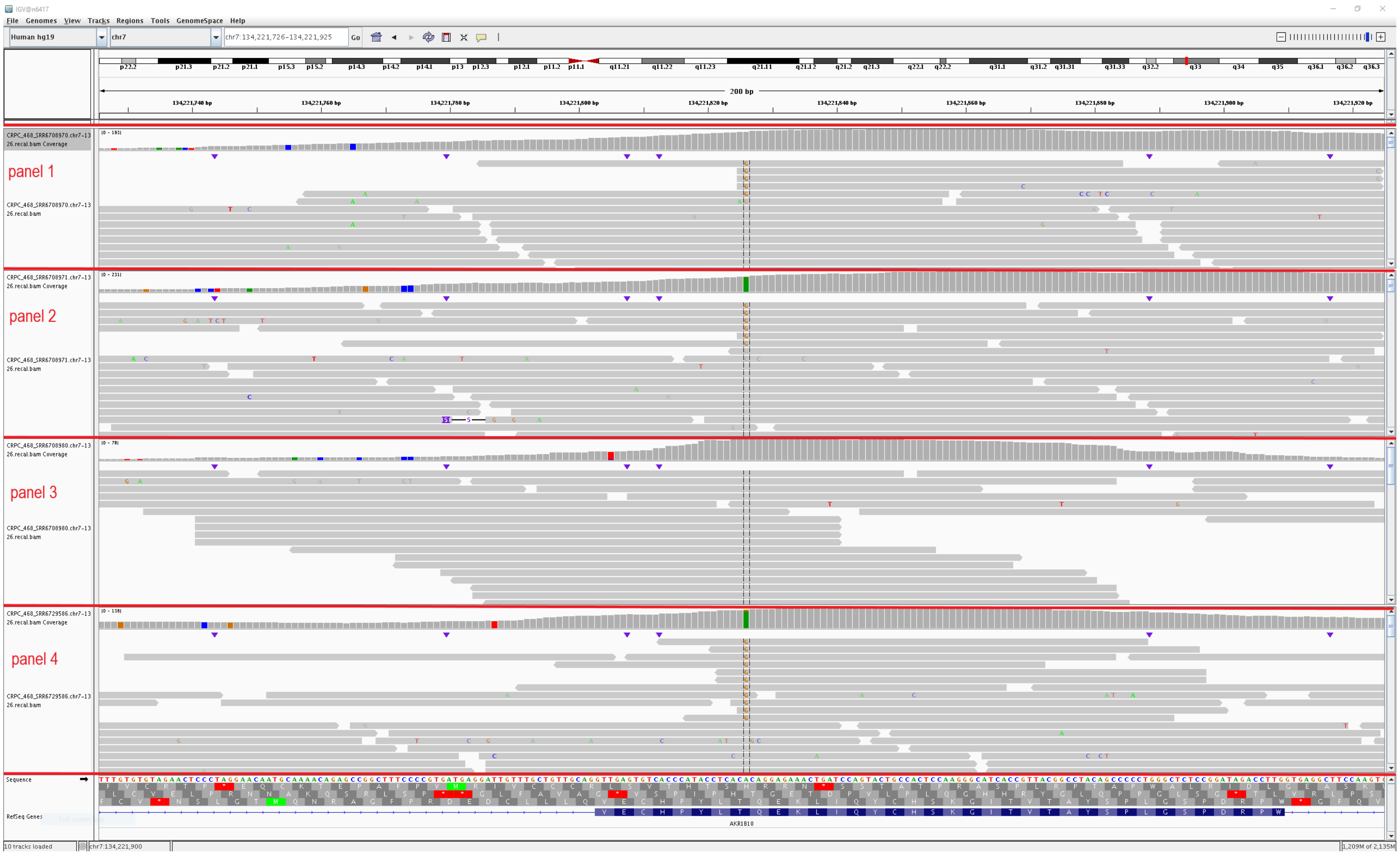
File Genomes View Tracks Regions Tools GenomeSpace Help
Human hg19
chr7
chr7:134,221,726-134,221,925
200 bp
CRPC_468_SRR6708970.chr7-13 26.recal.bam Coverage
panel 1
CRPC_468_SRR6708970.chr7-13 26.recal.bam
CRPC_468_SRR6708971.chr7-13 26.recal.bam Coverage
panel 2
CRPC_468_SRR6708971.chr7-13 26.recal.bam
CRPC_468_SRR6708980.chr7-13 26.recal.bam Coverage
panel 3
CRPC_468_SRR6708980.chr7-13 26.recal.bam
CRPC_468_SRR6729586.chr7-13 26.recal.bam Coverage
panel 4
CRPC_468_SRR6729586.chr7-13 26.recal.bam
Sequence
RefSeq Genes
AKR1B10
10 tracks loaded
chr7:134,221,900

Supplementary Fig. 12 | Case studies of mutations rescued from the standard clustered read position filter. Panel 1 and Panel 2 are plasma samples at two different time points. Panel 3 is blood normal sample. Panel 4 is tumor biopsy sample. The position between the two dashed vertical lines is the variant position. In the first plasma sample (panel 1) the variant base in three of five supporting reads clustered at the same location, so this position was filtered by the standard clustered read position. However, in the second plasma sample (panel 2) and the tumor biopsy sample (panel 4), there was no clustered read position event at this position, and it was detected as a mutation. Therefore this clustered read position event in panel 1 is likely due to non-random fragmentation other than misalignment.

Supplementary Tables

Performance metrics	cfSNV	MuTect	Strelka2	Strelka2 (filters disabled)
# predicted positives	386	129	149	1643
# true positives	386	129	149	190
# false positives	0	0	0	1453
Sensitivity = $\frac{\#\text{true positives}}{\#\text{ground-truth mutations}}$	64.0%	22.2%	25.6%	32.7%
Precision = $\frac{\#\text{true positives}}{\#\text{predicted positives}}$	100.0%	100.0%	100.0%	11.6%

Supplementary Table 1 | Performance of cfSNV on the simulation data with a high depth (around 2200x). The data contains 581 in silico inserted mutations simulated using BAMSurgeon as ground truth.

VAF for simulated mutations	# Ground-truth mutations	Sensitivity = $\frac{\#\text{true positives}}{\#\text{ground-truth mutations}}$ (# true positives)			
		cfSNV	MuTect	Strelka2	Strelka2 (filters disabled)
0.1%	116	39.7% (46)	5.2% (6)	0.9% (1)	2.6% (3)
0.3%	116	50.9% (59)	4.3% (5)	0.9% (1)	4.3% (5)
0.5%	58	53.4% (31)	3.4% (2)	0.0% (0)	3.4% (2)
0.8%	57	57.9% (33)	7.0% (4)	0.0% (0)	5.3% (3)
1%	59	74.6% (44)	11.9% (7)	0.0% (0)	3.4% (2)
3%	61	91.8% (56)	52.5% (32)	72.1% (44)	100.% (61)
5%	53	84.9% (45)	58.5% (31)	88.7% (47)	100.% (53)
8%	61	95.1% (58)	68.9% (42)	91.8% (56)	100.% (61)
Total	581	64.0% (386)	22.2% (129)	25.6% (149)	32.7% (190)

Supplementary Table 2 | Sensitivity of cfSNV on mutations at different VAFs on the simulation data with a high depth (around 2200x).

Sample ID	Time gap (days)	Truncal mutation confirmation rate	Branch mutation confirmation rate
CRPC_17.T1	24	95.5%	90.4%
CRPC_17.T2	24	100.0%	96.6%
CRPC_22.T1	21	100.0%	93.8%
CRPC_22.T2	21	94.9%	88.3%
CRPC_264.T1	28	100.0%	97.4%
CRPC_264.T2	28	100.0%	95.9%
CRPC_372.T1	62	100.0%	97.7%
CRPC_372.T2	62	100.0%	94.8%
CRPC_468.T1	14	99.0%	98.7%
CRPC_468.T2	14	99.0%	99.6%
CRPC_554.T1	138	100.0%	84.9%

CRPC_554.T2	138	96.3%	85.7%
MBC_191.T1	71	97.9%	93.1%
MBC_191.T2	71	72.7%	92.0%
MBC_284.T1	51	100.0%	92.2%
MBC_284.T2	51	100.0%	91.6%
MBC_288.T1	39	93.3%	97.8%
MBC_288.T2	39	97.4%	97.1%
MBC_295.T1	56	100.0%	91.6%
MBC_295.T2	56	92.9%	80.2%
MBC_303.T1	55	97.9%	90.9%
MBC_303.T2	55	100.0%	89.3%
MBC_307.T1	42	100.0%	96.3%
MBC_307.T2	42	96.3%	95.7%
MBC_313.T1	21	90.0%	91.5%
MBC_313.T2	21	92.9%	90.8%
MBC_318.T1	35	100.0%	90.6%
MBC_318.T2	35	100.0%	94.9%
MBC_325.T1	56	92.9%	78.8%
MBC_325.T2	56	100.0%	96.6%
MBC_331.T1	22	93.8%	97.4%
MBC_331.T2	22	96.4%	95.0%
MBC_339.T1	37	100.0%	97.7%
MBC_339.T2	37	100.0%	95.9%
MBC_349.T1	37	100.0%	97.7%
MBC_349.T2	37	100.0%	97.4%

Supplementary Table 3 | The plasma confirmation rate of the truncal and branch mutations in the validation patient cfDNA data and the time gap of the plasma collection between two time points.

Sample ID	Sequencing protocol	Total analyzed pairs	Overlapping pairs	Non-overlapping pairs	Overlapping rate
CRPC_161.T1	PE100	133713461	76706008	57007453	57.37%
CRPC_17.T2	PE100	112912255	68762359	44149896	60.90%
CRPC_17.T1	PE100	231379063	116648407	114730656	50.41%
CRPC_22.T2	PE100	69719765	40838217	28881548	58.57%
CRPC_22.T1	PE100	50922107	34962567	15959540	68.66%
CRPC_264.T1	PE100	94489440	49437464	45051976	52.32%
CRPC_264.T2	PE100	76285866	39931380	36354486	52.34%
CRPC_342.T1	PE100	77800434	39983987	37816447	51.39%
CRPC_362.T1	PE100	144733728	90831593	53902135	62.76%
CRPC_372.T1	PE100	95419408	46729963	48689445	48.97%
CRPC_372.T2	PE100	141469008	85545590	55923418	60.47%
CRPC_444.T1	PE100	99823352	58835498	40987854	58.94%
CRPC_463.T1	PE100	64410200	34965310	29444890	54.29%

CRPC_466.T1	PE100	86867442	40093027	46774415	46.15%
CRPC_468.T1	PE100	127076900	73725114	53351786	58.02%
CRPC_468.T2	PE100	125959241	69753527	56205714	55.38%
CRPC_525.T1	PE100	181507284	98384770	83122514	54.20%
CRPC_531.T1	PE100	191525002	110656948	80868054	57.78%
CRPC_554.T2	PE100	172555255	93394611	79160644	54.12%
CRPC_554.T1	PE100	115826043	69821170	46004873	60.28%
MBC_191.T1	PE100	49434221	37173220	12261001	75.20%
MBC_191.T2	PE100	104346049	58194183	46151866	55.77%
MBC_217.T1	PE100	45819537	31484308	14335229	68.71%
MBC_284.T1	PE100	187766846	115972627	71794219	61.76%
MBC_284.T2	PE100	105253472	57912040	47341432	55.02%
MBC_287.T1	PE100	72621076	47398698	25222378	65.27%
MBC_288.T1	PE100	109914978	60307802	49607176	54.87%
MBC_288.T2	PE100	71159260	49173622	21985638	69.10%
MBC_291.T1	PE100	125487153	79432588	46054565	63.30%
MBC_292.T1	PE100	169264847	109287196	59977651	64.57%
MBC_295.T1	PE100	72290837	40661840	31628997	56.25%
MBC_295.T2	PE100	95321907	45817182	49504725	48.07%
MBC_299.T1	PE100	145495818	89226402	56269416	61.33%
MBC_301.T1	PE100	139127700	80183853	58943847	57.63%
MBC_303.T1	PE100	172833875	103629229	69204646	59.96%
MBC_303.T2	PE100	70238383	39263198	30975185	55.90%
MBC_307.T1	PE100	107376735	63850659	43526076	59.46%
MBC_307.T2	PE100	147719840	83423006	64296834	56.47%
MBC_313.T1	PE100	129906737	75086942	54819795	57.80%
MBC_313.T2	PE100	145333019	83038161	62294858	57.14%
MBC_321.T1	PE100	233457402	125507698	107949704	53.76%
MBC_330.T1	PE100	131880037	83083092	48796945	63.00%
MBC_325.T1	PE100	113079532	67731787	45347745	59.90%
MBC_325.T2	PE100	139083004	94962794	44120210	68.28%
MBC_335.T1	PE100	176603512	113906872	62696640	64.50%
MBC_336.T1	PE100	188162046	143807711	44354335	76.43%
MBC_331.T2	PE100	106639523	60706708	45932815	56.93%
MBC_331.T1	PE100	153265273	92462423	60802850	60.33%
MBC_333.T1	PE100	295079422	221021645	74057777	74.90%
MBC_339.T1	PE100	122625928	72668646	49957282	59.26%
MBC_349.T2	PE100	123144300	87299997	35844303	70.89%
MBC_339.T2	PE100	118773552	68464597	50308955	57.64%
MBC_349.T1	PE100	135793407	96439191	39354216	71.02%
MBC_8.T1	PE100	102102243	61739698	40362545	60.47%
MBC_315.T1	PE100	106654039	63979866	42674173	59.99%
MBC_318.T1	PE100	97363967	65247299	32116668	67.01%
MBC_317.T1	PE100	89449800	53075788	36374012	59.34%
MBC_318.T2	PE100	109788289	73645143	36143146	67.08%
MBC_320.T1	PE100	169238177	94487319	74750858	55.83%
1129838	PE150	90493972	60668969	29825003	67.04%
3397799	PE150	98954989	62899357	36055632	63.56%

3736900	PE150	101233709	63795666	37438043	63.02%
4193384	PE150	92216457	56592576	35623881	61.37%
4258357	PE150	77145937	51554475	25591462	66.83%
4325774	PE150	112462632	69365854	43096778	61.68%
4492669	PE150	92722035	62999558	29722477	67.94%
4496246	PE150	53272682	36793304	16479378	69.07%
4514025	PE150	116818638	73998376	42820262	63.34%
4528560	PE150	73080877	47761989	25318888	65.35%
4532964	PE150	95269655	62118982	33150673	65.20%
4536877	PE150	84576575	53857762	30718813	63.68%
4545410	PE150	105795081	72662041	33133040	68.68%
4561279	PE150	125089513	80524552	44564961	64.37%
4563728	PE150	31166243	22383526	8782717	71.82%
4583975	PE150	117726231	69731179	47995052	59.23%
4599369	PE150	100124756	63787421	36337335	63.71%
2163573	PE150	94058875	59201901	34856974	62.94%
4650336	PE150	92495802	54860077	37635725	59.31%
2510880	PE150	70282777	41196303	29086474	58.62%
4390360	PE150	84718207	53665469	31052738	63.35%
4471067	PE150	84266052	47593768	36672284	56.48%
4566326	PE150	97320644	55114291	42206353	56.63%
4582920	PE150	59732944	33126627	26606317	55.46%
4612584	PE150	95861797	57164638	38697159	59.63%
4562675	PE150	87382996	49998703	37384293	57.22%
2450596	PE150	81592146	49147981	32444165	60.24%
4335068	PE150	87083450	47799248	39284202	54.89%
4526552	PE150	93363300	63106874	30256426	67.59%
4411770	PE150	56819893	37203845	19616048	65.48%
4580642	PE150	113976400	76878551	37097849	67.45%
4637842	PE150	69840351	44686904	25153447	63.98%

Supplementary Table 4 | Statistics of overlapping read pairs in the cfDNA samples.

Filter	Description and default thresholds	Pass	Hold
Strand bias	Removes false positives caused by context-specific or systematic sequencing errors. These are recognized by observing an abnormal number of variant alleles in a single direction of reads. We test for strand bias by calculating the binomial probability that variant alleles are only observed in a single direction of reads. The parameter used in the binomial distribution is the strand ratio, calculated from reference supporting alleles. Candidates are rejected if the binomial probability is less than 0.05. This threshold is equivalent to saying that when variant alleles are observed from both directions, the ratio between forward variant alleles and reverse variant alleles must be in the range [7, 1/7] to pass this filter.	pass	pass

Variant frequency	Remove false positives caused by tri-allelic sites or random sequencing errors. We compare the number of variant supporting reads and the number of non-germline reads. If the fraction of variant supporting reads in all non-germline reads is less than 0.8, then the candidate is rejected.	pass	pass
Mapping quality	Remove false positives caused by context-specific or location-specific misalignments, so that reads aligned to the candidate site have a lower mapping quality in general. Candidates always pass the filter when there are enough reads (n > 20) with high mapping quality (phred > 10). Otherwise, candidates are rejected if there are more than 3 reads with low mapping quality (phred < 5) and the number of total reads is less than 20. Candidates are also rejected if the fraction of reads with low mapping quality is greater than 0.4, or if the median mapping quality at the position is low (phred < 10). Finally, candidates meeting none of these criteria pass the filter.	pass	pass
Variant allele mapping quality	Remove false positives caused by misalignment, where reads aligned to the candidate have a lower mapping quality in general. Candidates pass the filter directly when there are enough reads (n > 3) with high mapping quality (phred > 10). Candidates are rejected if there are more than 3 reads with low mapping quality (phred < 5) and the number of total reads is less than 20. Candidates are also rejected if the fraction of reads with low mapping quality is greater than 0.4, or if median mapping quality at the position is low (phred < 10). Otherwise, candidates pass the filter.	pass	pass
Variant base quality	Remove false positives caused by incorrect base calls. Candidates are rejected when the median base call error probability of variants is more than 7 times the median base call error probability of reference bases. Candidates are also rejected if the number of high-quality variant bases (phred > 23) is fewer than 3.	pass	pass
Supporting fragments	Mark candidates with strong evidence. Candidates with more than three supporting reads are marked as having strong evidence.	pass	-
Tumor coverage	Remove false positives caused by inadequate sequencing. Candidates are rejected if they have ≤ 10x coverage in plasma. Candidates are marked as having a low-confidence VAF if they have coverage > 10x and ≤ 50x in plasma.	pass	pass
Normal coverage	Remove false positives caused by inadequate sequencing of the matched germline blood sample. Candidates are rejected if their coverage is ≤ 7 in the germline blood sample.	pass	pass
Nearby repeats	Remove false positives caused by misalignment of nearby repeats. Candidates are rejected if they are within a repeat region annotated by RepeatMasker.	pass	pass
Nearby indels	Remove false positives caused by misalignment of nearby indels. Candidates are rejected if they are within a distance of 5 base pairs from an indel. Indels are marked by the alignment tool (BWA), and collected for use in this filter if there are ≥ 3 reads supporting an indel at the position or if the fraction of reads supporting an indel is greater than 0.02.	pass	pass

Binomial VAF test	Remove false positives with low confidence given the current global tumor fraction. Candidates are rejected if the binomial probability of observing the number of variant supporting reads is less than 0.1.	pass	-
Public databases	Remove germline variants by rejecting candidates present in a public germline database (dbSNP).	pass	pass
Co-occurrence of candidates	Remove false positives associated with misalignment. Candidates are rejected if they always co-occur with other candidates on the variant supporting reads, or their position on the reads are always the same.	pass	pass

Supplementary Table 5 | Description of site-level post-filtration criteria and thresholds.

Category from iterative detection	Passing criteria
pass	number of variant supporting reads > 5
pass	number of variant supporting reads > 3 and binomial probability of observing more variant supporting reads given the tumor fraction < 0.6
hold	number of variant supporting reads > 12
hold	number of variant supporting reads > 5 and binomial probability of observing more variant supporting reads given the tumor fraction < 0.6

Supplementary Table 6 | Description of read-level post-filtration criteria and thresholds.

Feature description	Feature type	Non-Overlapping model	Overlapping model
base call in a 7-bp window centered on the query site from the read	categorical	yes	yes
base quality in a 7-bp window centered on the query site from the read	numerical	yes	yes
CIGAR information in a 7-bp window centered on the query site from the read	categorical	yes	yes
Occurrence CIGAR operators from the read	Boolean	yes	yes
mapping quality of the read pair	numerical	yes	yes
distance to the nearest indel on the read pair	numerical	yes	yes
whether the query site was contained in a homopolymer with size >= 5	Boolean	yes	yes
insertion sizes of the read pair	numerical	yes	yes
mapping flags of the read and the mate	categorical	yes	yes
genome sequence in a 7-bp window centered on the query site	categorical	yes	yes
base call in a 7-bp window centered on the query site from the mate	categorical	no	yes

base quality in a 7-bp window centered on the query site from the mate	numerical	no	yes
CIGAR information in a 7-bp window centered on the query site from the mate	categorical	no	yes

Supplementary Table 7 | Extracted features from read pairs for the random forest models. The column non-overlapping model indicates which features are used in the random forest model for purifying non-overlapping read pairs. The column overlapping model indicates which features are used in the model for overlapping read pairs.

	Sites	Total read pairs	Overlapping read pairs	Non-overlapping read pairs
Variant SRR6708941	33567	4168752	735310	3430446
Variant SRR6708920	33567	574143	109646	464497
Error SRR6708941	37992	36238	14316	21922
Error SRR6708920	2996	2386	1046	1340

Supplementary Table 8 | Training data for the random forest model. Reads from two experiments of the same plasma sample were labeled as containing true variants or sequencing errors. Reads from SRR6708941 were used for training, while reads from SRR6708920 were only used for validating the model.

ID		Error		Variant		
Patient ID	Sample ID	No Overlap	Overlap	No Overlap	Overlap	Total
CRPC_17	SRR6708976	53265	16702	2732201	626848	3429016
CRPC_17	SRR6708977	482728	177841	1800489	421860	2882918
CRPC_22	SRR6708978	83022	24613	1940412	407109	2455156
CRPC_22	SRR6708979	36329	9603	1268275	301515	1615722
CRPC_264	SRR6708961	46548	21471	2198144	591466	2857629
CRPC_264	SRR6708962	19802	9958	1701362	452894	2184016
CRPC_372	SRR6708965	190221	80599	2285711	572887	3129418
CRPC_372	SRR6708966	10453	6778	3397767	840300	4255298
CRPC_468	SRR6708970	26631	18890	3513882	903369	4462772
CRPC_468	SRR6708971	36241	25120	3483517	900246	4445124
CRPC_554	SRR6708974	664047	223230	5270051	1000723	7158051
CRPC_554	SRR6708975	77130	24954	3780842	719078	4602004
MBC_191	SRR6708921	215	143	1251002	414562	1665922
MBC_191	SRR6708922	1067895	538961	2594943	827221	5029020
MBC_284	SRR6708924	23625	11925	4893262	1183064	6111876
MBC_284	SRR6708925	144682	59011	2844469	780684	3828846
MBC_288	SRR6708927	737942	339409	2959737	971247	5008335
MBC_288	SRR6708928	431	527	1936307	757024	2694289
MBC_295	SRR6708931	105029	65050	2210291	466676	2847046
MBC_295	SRR6708932	2156879	1106220	2902209	702418	6867726
MBC_303	SRR6708935	30453	17647	5086895	989472	6124467
MBC_303	SRR6708936	189886	118493	2108430	550301	2967110

MBC_307	SRR6708937	12880	7310	3282372	682269	3984831
MBC_307	SRR6708938	1009750	418810	3806616	930340	6165516
MBC_313	SRR6708939	18583	9892	3847262	855336	4731073
MBC_313	SRR6708940	1600957	802650	3554513	1080191	7038311
MBC_318	SRR6708943	8089	7567	3196004	820666	4032326
MBC_318	SRR6708944	504118	436302	2703510	1029187	4673117
MBC_325	SRR6708947	372052	267233	2888921	962712	4490918
MBC_325	SRR6708948	88376	57088	3338437	945579	4429480
MBC_331	SRR6708950	478302	281906	4280243	1030174	6070625
MBC_331	SRR6708951	924486	609734	2889249	748642	5172111
MBC_339	SRR6708955	189355	67166	3900113	786977	4943611
MBC_339	SRR6708956	306985	107457	3673671	728964	4817077
MBC_349	SRR6708957	170348	58320	3870204	883532	4982404
MBC_349	SRR6708958	161196	55983	3739991	887266	4844436

Supplementary Table 9 | Sample IDs and number of testing reads extracted. Patient IDs follow the naming convention in [1], while sample IDs are the SRA accession IDs of the sample.

Supplementary References

[1] Adalsteinsson, Viktor A., et al. Scalable whole-exome sequencing of cell-free DNA reveals high concordance with metastatic tumors. *Nature communications* 8.1 (2017): 1324.
